# Reciprocal amplification of caspase-3 activity by nuclear export of a putative human RNA-modifying protein, PUS10 during TRAIL-induced apoptosis

**DOI:** 10.1038/cddis.2017.476

**Published:** 2017-10-05

**Authors:** Sujata Jana, Andrew C Hsieh, Ramesh Gupta

**Affiliations:** 1 Department of Biochemistry and Molecular Biology, Southern Illinois University, Carbondale, IL 62901-4413, USA; 2 Division of Human Biology, Fred Hutchinson Cancer Research Center, Seattle, WA 98109, USA; 3 School of Medicine, University of Washington, Seattle, WA 98195, USA

## Abstract

Pus10 is a pseudouridine synthase present in Archaea and Eukarya, but not in Bacteria and yeast. It has been suggested that the human *PUS10* (*DOBI*) gene is needed during TRAIL-induced apoptosis. We analyzed the role of PUS10 in TRAIL-induced apoptosis by immunofluorescence, immunoblotting and several indicators of apoptosis. We examined several TRAIL-sensitive cell lines and we also examined some resistant cell lines after treatment with cycloheximide. PUS10 is mainly present in the nucleus. Early during apoptosis, PUS10 translocates to mitochondria via CRM1-mediated export with the concurrent release of cytochrome c and SMAC. Caspase-3 is required for PUS10 translocation, which reciprocally amplifies the activity of caspase-3 through the intrinsic/mitochondrial pathway. This suggests that in addition to cytoplasmic factors, nuclear factors also have a direct role in the major apoptosis pathways. However, p53 is not involved in TRAIL-induced PUS10 movement. The caspase-3-mediated movement of PUS10 and the release of mitochondrial contents enhancing caspase-3 activity creates a feedback amplification loop for caspase-3 action. Therefore, any defect in the movement or interactions of PUS10 would reduce the TRAIL sensitivity of tumor cells.

Apoptosis is a genetically determined mode of cell death that relies on energy-dependent cascade of caspase-mediated molecular events.^1, 2, 3, 4, 5^ It is important for natural cell turnover during development and aging, and in the elimination of virus-infected and damaged cells. It is of clinical relevance for cancer treatment by irradiation or drugs. There are two major pathways of apoptosis: extrinsic (death receptor) and intrinsic (mitochondrial). Binding of a ligand, such as TRAIL, to its specific death receptors on the cell surface initiates the extrinsic pathway, mainly by activation of caspase-8, whereas ‘stress signals’ or internal factors initiate the intrinsic pathway through activation of pro-apototic proteins, for example, BAK and BAX. There is also ‘cross-talk’ between the two pathways via activation of BID.

TRAIL, a member of the TNF superfamily, is naturally involved in tumor surveillance.^6^ Recombinant TRAIL can induce apoptosis in several types of cancer cells, but leaves normal cells unaffected.^3, 7, 8^ However, certain cancer cells are either resistant to TRAIL or develop resistance during treatment. This is mainly due to mutations of various factors involved in apoptosis.^3, 8, 9^ Binding of TRAIL to the death receptors DR4 and DR5 recruits FADD and procaspase-8, which form the death-inducing silencing complex (DISC), resulting in caspase-8 activation.^10^ Based on the levels of DISC and caspase-8, cells are categorized into two types.^1, 3, 4, 7, 11^ In type I cells, the levels of activated caspase-8 are high and are sufficient to activate the effector caspases-3, -6 and -7. In type II cells, caspase-8 also cleaves BID to its truncated form, tBID. The tBID releases cytochrome c, SMAC/DIABLO, OMNI and other factors from the mitochondria. The released cytochrome c forms the apoptosome complex with APAF-1 and procaspase-9. This results in active caspase-9, which in turn activates the effector caspases, thus linking the extrinsic and intrinsic pathways. The intrinsic pathway can also be initiated, independent of BID, by the oligomerization of pro-apoptotic proteins, for example, BAK, BAX and so on, which permeabilize the outer mitochondrial membrane and release cytochrome c, SMAC, OMNI and so on.^1, 4, 12^ The effector caspases cleave several proteins that lead to DNA fragmentation, chromatin condensation, cell membrane blebbing, cell shrinkage, formation of apoptotic bodies and cell death.^7^ The activities of some caspases are redundant and overlap, for example, initiator caspase-8 with caspase-10, and effector caspase-3 with caspase-6 and -7. The effector caspases also regulate upstream events via a feedback amplification loop involving mitochondria.^1, 13^ SMAC released from mitochondria during apoptosis inactivates XIAP, an inhibitor of caspases-3, -7 and -9, further activating these caspases.^1, 4, 13^

RNAi-mediated phenotypic screen of HeLa cells suggested that the human *PUS10* (*DOBI*, *FLJ32312*) is required for the TRAIL-induced apoptotic signal to progress through the intrinsic pathway.^6^ The siRNA against *PUS10* inhibited TRAIL-induced caspase-3/7 activity and additionally mimicked the results of siRNA against *BID* in preventing caspase-9 processing without affecting caspase-8 or caspase-3 cleavage. Furthermore, reduced *PUS10* activity prevented TRAIL-induced cytochrome c release from the mitochondria.

The crystal structure of human PUS10 (HuP10) shows that the protein has two domains: an *N*-terminal (Met1-His285) THUMP-containing domain and a *C*-terminal (Gly286-Asp528) pseudouridine (Ψ) synthase domain.^14^ The *C*-terminal domain contains the full set of conserved Ψ synthase active site residues, suggesting that HuP10 may function as a Ψ synthase. Ψ is the most common post-transcriptionally modified residue in RNA^15^ and may be produced by members of six different families of Ψ synthases.^16^ So far, Pus10 is the only known member of its family of Ψ synthases. *Pus10* (*PsuX*) homologs are present in Archaea and most Eukarya, but not in Bacteria or yeast.^14, 17, 18^ *Pus10* is an essential gene in Archaea.^19^ Archaeal Pus10 produces Ψ54 and Ψ55 in tRNAs,^20, 21, 22^ but there is no published report indicating whether HuP10 has any Ψ synthase activity.

The purpose of this study was to determine the role of HuP10 in TRAIL-induced apoptosis. We observed that HuP10 is exported from the nucleus to the mitochondria in the early stages of TRAIL-induced apoptosis, with the concurrent release of cytochrome c and SMAC. This export of HuP10 is CRM1-mediated and requires active caspase-3. In turn, the movement of HuP10 reciprocally amplifies caspase-3 activity, suggesting a feedback loop is involved.

## Results

### HuP10 translocates from nucleus to mitochondria during TRAIL-induced apoptosis

**Figure 1:**
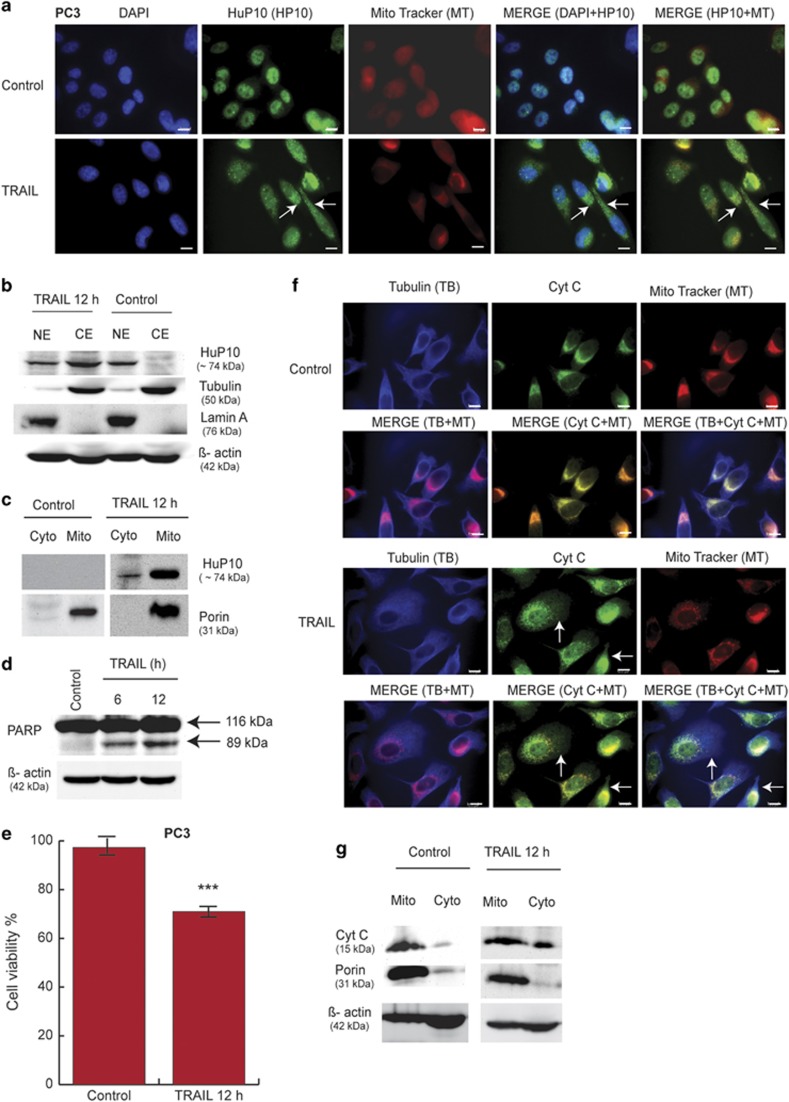
Movement of HuP10 from nucleus to mitochondria after TRAIL treatment of PC3 cells. (**a**) PC3 cells cultured on coverslips were treated with TRAIL (0.5 *μ*g/ml, 12 h). Both treated and untreated (Control) cells were incubated with Mito Tracker dye (red) to stain mitochondria, followed by IF using anti-HuP10 antibody (green) and nuclear staining by DAPI (blue). Anti-HuP10 antibody recognizes HuP10 in the nucleus and an unknown protein present in both nucleus and cytoplasm (see Supplementary Figures S1D–S1E), which causes light green coloration of the cytoplasm. Arrows indicate HuP10 in cytoplasm, overlapping the mitochondria. Bars=10 *μ*m. (**b**) Nuclear (NE) and cytoplasmic (CE) fractions of untreated (Control) and TRAIL-treated cells were analyzed by IB using anti-HuP10 antibody. Anti-lamin A and anti-tubulin antibodies were used to check the purity of NE and CE, respectively. *β*-actin was used as a loading control. (**c**) IB analyses of mitochondrial and mitochondria-depleted cytoplasmic fraction of TRAIL-treated and untreated cells using anti-HuP10 antibody. Porin is the mitochondrial marker. (**d**) IB analysis of lysates of cells treated with TRAIL for 6 h or 12 h, and controls, using anti-PARP antibody to determine PARP cleavage. *β*-actin was used as loading control. See also Supplementary Figure S4A. (**e**) Cell viability was determined by the MTT assay after TRAIL treatment. Values are mean±S.E. (*n*=3). ***=*P*<0.001 *versus* control. (**f**) Double IF assay of TRAIL-treated (12 h) and control cells with anti-cytochrome c (green) and anti-tubulin (blue) antibodies (cytoplasmic marker). Mitochondria were stained with Mito Tracker dye (red). Arrows indicate the small amount of cytochrome c released from mitochondria into the cytoplasm (overlapping tubulin distribution) in TRAIL-treated cells. Bars=10 *μ*m. (**g**) IB analyses of mitochondrial and mitochondria-depleted cytoplasmic fractions of TRAIL-treated and control cells using anti-cytochrome c antibody. There is more cytochrome c relative to *β*-actin in cytoplasm of TRAIL-treated cells than the control cells. Porin is a mitochondrial marker

We used PC3 (prostate cancer) cells, which are p53 null,^23^ to determine the role of HuP10 in TRAIL-induced apoptosis. This was done to avoid any effects of p53 in apoptosis. Both immunofluorescence (IF) of cells and immunoblot (IB) analyses of nuclear and cytoplasmic fractions using a commercially available anti-HuP10 antibody determined that HuP10 is normally present in the nucleus (controls in Figures 1a and b). (This antibody recognizes HuP10 in both IB and IF analyses; see Supplementary Figures S1C–S1I) The granular appearance of the signal suggests that HuP10 may be concentrated in certain areas within the nuclei, although it does not seem to be present in the nucleoli. A search for the Nuclear Localization Signal (http://nls-mapper.iab.keio.ac.jp/cgi-bin/NLS_Mapper_form.cgi^24^) in the HuP10 sequence predicted a signal at aa positions 64–74 of the protein (Supplementary Figure S1A), which is conserved in other mammalian homologs (Supplementary Figure S1B). The published crystal structure of HuP10^14^ does not contain this signal motif, presumably as residues 63–75 were cleaved by limited proteolysis before crystallization.

TRAIL treatment of PC3 cells for 12 h showed that HuP10 moved from the nucleus to the cytoplasm. In particular, we found that HuP10 specifically localized to the mitochondria, both by IF and IB (Figure 1a–c).

HuP10 translocation appeared to be associated with the early stages of apoptosis because significant morphological changes synonymous with the later stages of programed cell death were not observed after 12 h of TRAIL exposure. However, our observations by PARP cleavage, cell viability, cytochrome c release and Annexin V flow cytometry analyses indicated that the cells were clearly initiating the apoptosis program. In particular, PARP cleavage increased in a time-dependent manner (Figure 1d and Supplementary Figure S4A). MTT assays showed that the cell viability decreased after 12 h of TRAIL treatment (Figure 1e). TRAIL treatment induced cytochrome c release from the mitochondria into the cytoplasm (Figure 1g, also shown in Figure 7a and b), overlapping with the distribution of tubulin in the cytoplasm (Figure 1f).

To see if HuP10 movement during TRAIL-induced apoptosis was a general phenomenon, we tested several other cell lines that are either sensitive to or can be sensitized to TRAIL. IF analyses of both MDA-MB-231 (breast cancer) and RH30 (alveolar rhabdomyosarcoma) cell lines showed movement of HuP10 from the nucleus to the mitochondria (Supplementary Figures S2A–B), during TRAIL-induced apoptosis, as determined by PARP cleavage (Supplementary Figures S2C–D and S4C–D). These cell lines, like PC3 are TRAIL-sensitive.^25, 26, 27^ HeLa and LNCaP (prostate cancer) cells have limited sensitivity to TRAIL^8, 28^ but their sensitivity can be enhanced by cycloheximide (CHX).^29, 30^ As expected, treatment of these cells with TRAIL alone produced very little PARP cleavage (Supplementary Figures S3A, S3D and S4E–F) when compared with TRAIL-sensitive cells (Figure 1d, and Supplementary Figures S2C–D). However, simultaneous treatment of these cells with CHX and TRAIL showed increased PARP cleavage compared with TRAIL alone (Supplementary Figures S3A and S3D and S4E–F). Similarly, viability was significantly reduced only when the HeLa and LNCaP cells were simultaneously treated with CHX and TRAIL (Supplementary Figures S3B and S3E). IF data showed HuP10 translocation only occurred in these cells when CHX accompanied TRAIL treatment (Supplementary Figures S3C and S3F). Overall, these results demonstrate that HuP10 is normally present in the nucleus and moves from there into the mitochondria early during TRAIL-induced apoptosis.

### The CRM1-mediated nuclear export pathway is involved in HuP10 movement

**Figure 2:**
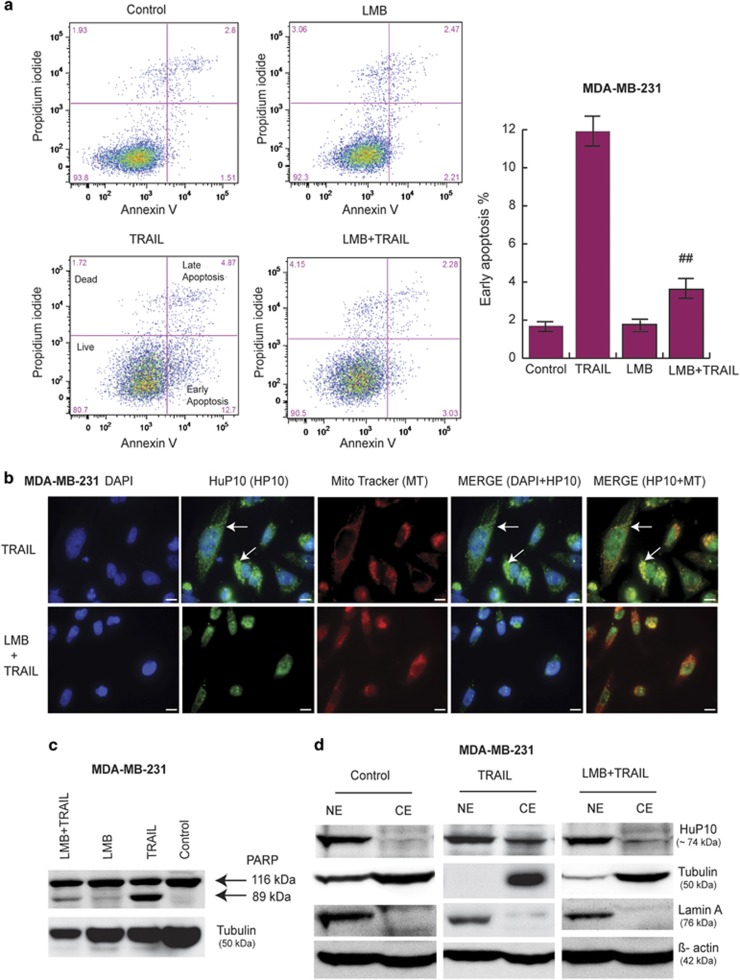
Involvement of CRM1-mediated nuclear export pathway in movement of HuP10. (**a**) PI/Annexin V analysis of apoptosis in MDA-MB-231 cells after treatment with only TRAIL (0.5 *μ*g/ml) for 3 h, only LMB (5 ng/ml) for 5 h, or with LMB (5 ng/ml) for 2 h followed by TRAIL (plus LMB) for 3 h. The flow cytometry profile represents Annexin V and Propidium iodide staining along the *X* and *Y* axis, respectively. The values shown in the lower left, lower right, upper right and upper left quadrants of each panel represent the percentage of live, early apoptotic, late apoptotic and dead cells, respectively. The bar graph shows early apoptotic cells (%). Values are mean±S.E. (*n*=3). ##=p 0.01 *versus* TRAIL. (**b**) IF analyses of MDA-MB-231 cells treated with TRAIL (0.5 *μ*g/ml) for 3 h (upper panels) as in Supplementary Figure S2A and with LMB (5 ng/ml) for 2 h followed by TRAIL (plus LMB) for 3 h (lower panels). Staining as in Figure 1a. Arrows in TRAIL alone treatment indicate HuP10 in the cytoplasm. Bars=10 *μ*m. See also Supplementary Figure S5B. (**c**) IB analysis showing decrease in TRAIL-induced PARP cleavage in MDA-MB-231 when the cells were treated with LMB and then by TRAIL as in (**a**) and (**b**). Tubulin is a loading control. See also Supplementary Figure S4B. (**d**) Nuclear (NE) and cytoplasmic (CE) fractions of untreated, TRAIL-treated and LMB+TRAIL-treated cells were analyzed by IB using anti-HuP10 antibody. Lamin A and tubulin are nuclear and cytoplasmic markers, respectively. *β*-actin was used as a loading control. Much less HuP10 is present in the cytoplasm in LMB+TRAIL treatment then TRAIL alone treatment

CRM1, also called Exportin-1, is a major export protein involved in transport of RNAs, proteins and ribonucleoprotein complexes from the nucleus to the cytoplasm.^23^ Leptomycin B (LMB) is an inhibitor of CRM1.^31^ We could not use LMB with PC3 cells because LMB is toxic for PC3 cells.^23^ We confirmed this toxicity by a cell viability assay (Supplementary Figure S5A). As such, we used MDA-MB-231 cells, which we found can tolerate LMB (Figure 2a and Supplementary Figure S5B), to determine if CRM1 mediates the transport of HuP10 from the nucleus to the mitochondria. TRAIL-induced apoptosis was reduced in these cells in the presence of LMB (Figure 2a). Transfer of HuP10 from the nucleus to the cytoplasm was also blocked in these cells in the presence of LMB (Figure 2b). LMB also significantly inhibited TRAIL-induced PARP cleavage (Figure 2c and Supplementary Figure S4B). IB analysis also showed that HuP10 was restricted to the nucleus after TRAIL treatment, if the cells were pre-treated with LMB (Figure 2d). These results suggest that CRM1 is needed for the nuclear export of HuP10 and that this transfer of HuP10, a nuclear protein, enhances apoptosis.

### Caspase-3 is involved in TRAIL-induced translocation of HuP10

**Figure 3:**
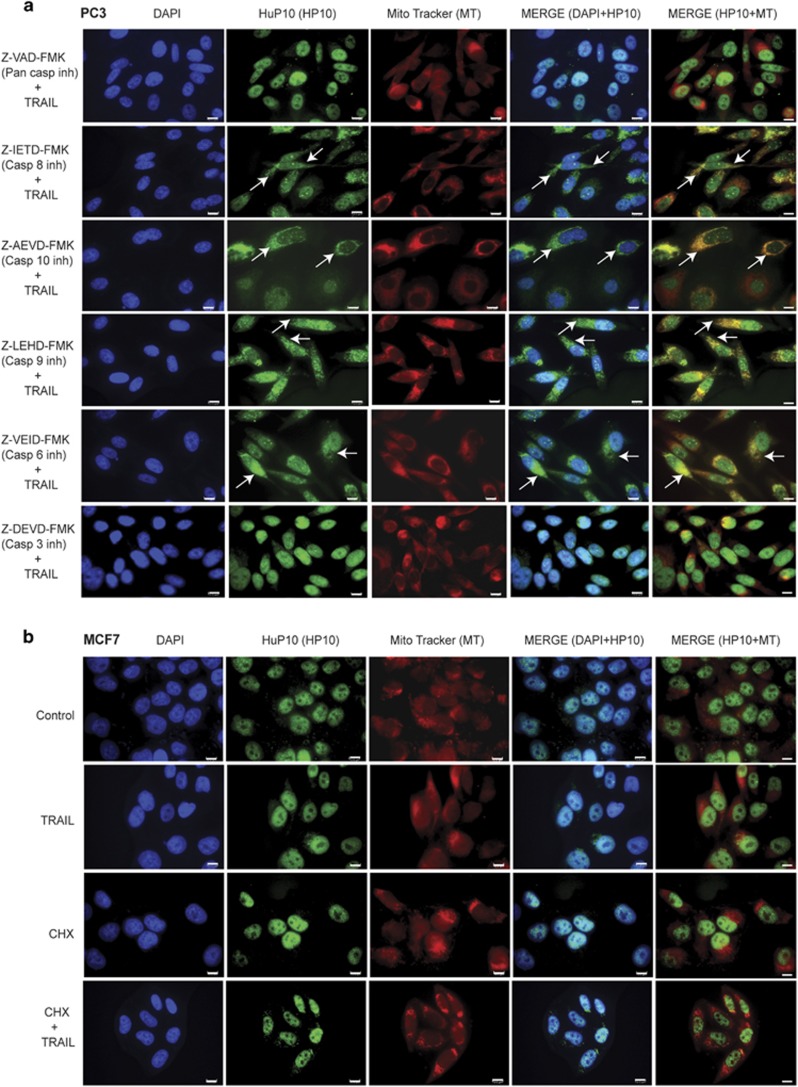
Involvement of caspase-3 in TRAIL-induced movement of HuP10. (**a**) IF analyses of PC3 cells grown on coverslips at 80% confluency were pre-treated separately with a pan-caspase inhibitor (Z-VAD-FMK, 50 *μ*M) and individual inhibitors of caspase-8 (Z-IETD-FMK, 40 *μ*M), -10 (Z-AEVD-FMK, 40 *μ*M), -9 (Z-LEHD-FMK, 40 *μ*M), -6 (Z-VEID-FMK, 40 *μ*M), and -3 (Z-DEVD-FMK, 40 *μ*M) for 3 h followed by 12 h TRAIL treatment (0.5 *μ*g/ml; plus inhibitors). Staining as in Figure 1a. Most individual inhibitors do not block transfer of HuP10 to cytoplasm (arrows). Bars=10 *μ*m. See also Supplementary Figure S6A. (**b**) IF analyses of MCF7 cells after simultaneous treatment with CHX (0.5 *μ*M) and TRAIL (0.5 *μ*g/ml) alone or together for 12 h. Untreated cells are control. Staining as in Figure 1a. Bars=10 *μ*m. See also Supplementary Figure S7

We treated PC3 cells with Z-VAD-FMK, a pan-caspase inhibitor to determine whether any caspase is involved in the TRAIL-induced translocation of HuP10. HuP10 was restricted to the nucleus in this case (Figure 3a, first row), suggesting a role of one or more caspases in its translocation. Therefore, to pinpoint the specific caspase, we treated PC3 cells with inhibitors of individual caspases. Inhibitors of initiator caspase-8, -9 and -10 did not block TRAIL-induced movement of HuP10 (Figure 3a), suggesting that these caspases are not essential for HuP10 movement. As initiator caspases have overlapping activities, we also used combinations of inhibitors for two initiator caspases at a time before TRAIL treatment. None of these combinations restricted HuP10 movement (Supplementary Figure S6A). Next, we tested inhibitors of executioner caspase-3 and -6 (An inhibitor of caspase-7 only is not commercially available). The caspase-6 inhibitor did not block HuP10 movement (Figure 3a). However, the caspase-3 inhibitor blocked HuP10 movement just like the pan-caspase inhibitor (compare first and last rows of Figure 3a), which was also confirmed by IB analysis (Supplementary Figure S6D). PARP cleavage and caspase-3 activity assays confirmed that all these inhibitors were functional under our experimental conditions (Supplementary Figures S6B–C).

We used MCF7 (breast cancer) cells, which lack caspase-3 activity,^32, 33^ to further investigate the effect of caspase-3 on HuP10 translocation. MCF7 cells are TRAIL-resistant,^34^ but can be sensitized to TRAIL by CHX.^35^ Apoptosis in MCF7 cells is suggested to proceed via sequential activation of caspase-9, -7 and -6, with caspase-7 cleaving PARP.^32, 33^ We confirmed that MCF7 cells undergo TRAIL-induced apoptosis in the presence of CHX by cell viability and PARP cleavage assays (Supplementary Figures S7A–B and S4H). Although cleaved PARP was present after 6 h of CHX+TRAIL treatment (Supplementary Figure S7B), we detected no cleaved PARP and very little full sized PARP after 12 h. HuP10 did not move out of the nucleus in response to TRAIL alone or to TRAIL+CHX (Figure 3b). IB assays of nuclear and cytoplasmic fractions of TRAIL+CHX-treated cells also showed that HuP10 remains within the nucleus (Supplementary Figure S7C).

**Figure 4:**
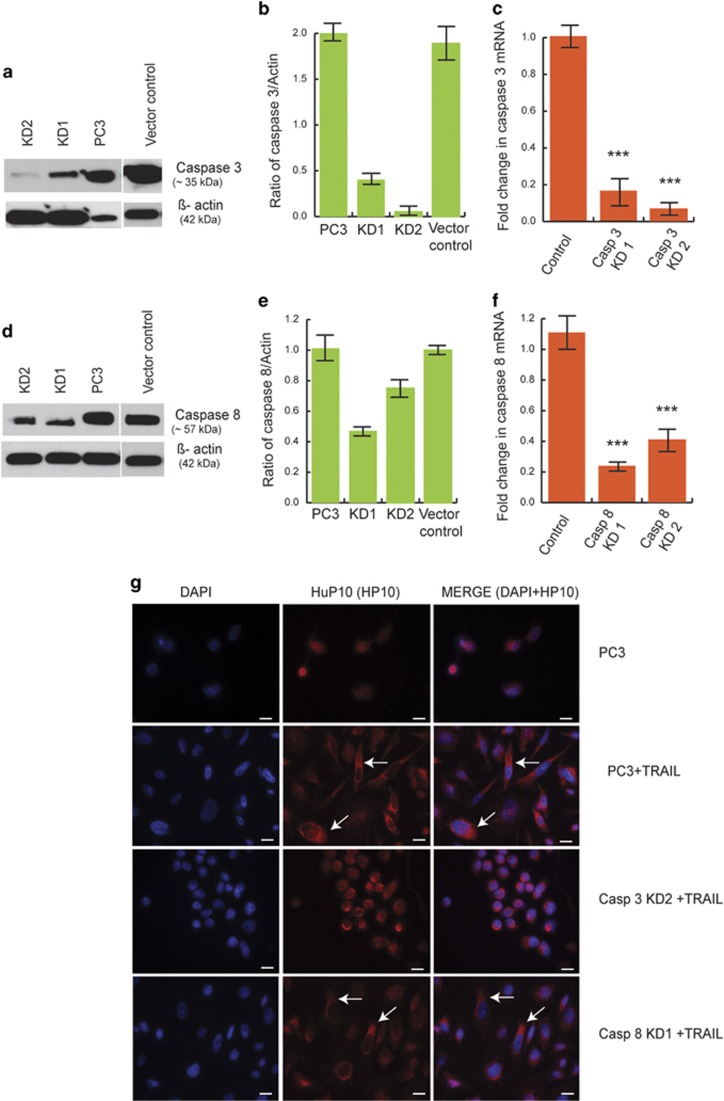
Knockdown of caspase-3, but not of caspase-8 restricts TRAIL-induced movement of HuP10. (**a**) IB analysis of cell lysates of two independent Caspase-3 KD PC3 clones (KD1 and KD2) showed the reduced amount of caspase-3 in the cells. Lysates of normal PC3 and empty vector transfected PC3 cells are used as controls. *β*-actin is the loading control. (**b**) Densitometric analysis of two independent blots as in (**a**), showing ratio of caspase-3 to *β*-actin. (**c**) qPCR was used to compare the amount of caspase-3 mRNA in PC3 clone KD1 and KD2 with normal PC3 cells as control to confirm the knockdown of caspase-3. Results are normalized to actin mRNA. Values are mean±S.E. (*n*=4). ***=*P*<0.001 *versus* control. (**d**) IB analysis of cell lysates of two independent Caspase-8 KD PC3 clones (KD1 and KD2) showed the reduced amount of caspase-8 in the cells. Lysates of normal PC3 and empty vector transfected PC3 cells are used as controls. *β*-actin is the loading control. (**e**) Densitometric analysis of two independent blots as in (**d**), showing ratio of caspase-8 to *β*-actin. (**f**) qPCR was used to compare the amount of caspase-8 mRNA in PC3 clone KD1 and KD2 with normal PC3 cells as control to confirm the knockdown of caspase-8. Results are normalized to actin mRNA. Values are mean±S.E. (*n*=4). ***=*P*<0.001 *versus* control. (**g**) Caspase-3 and caspase-8 knockdown PC3 cells cultured on coverslips were treated with TRAIL (0.5 *μ*g/ml, 12 h). The IF was done using anti-HuP10 antibody (red) and nuclear staining by DAPI (blue). As the caspase-3 and caspase-8 shRNA construct contains a green GFP marker, Alexa Fluor 568 (red) secondary antibody was used here to detect HuP10. Arrows indicate HuP10 in cytoplasm. Bars=10 *μ*m

To further prove that caspase-3 and not the extrinsic pathway-specific caspase-8 is involved in HuP10 translocation, we knocked down both of these caspases in PC3 cells by shRNA. We observed decreased levels of caspase-3 and -8 protein and mRNA upon lentiviral infection of specific shRNA constructs (Figure 4a–f). As expected, HuP10 did not exit the nucleus upon caspase-3 knockdown (KD), whereas it did exit in the case of caspase-8 KD (Figure 4g). These findings corroborate what we observed in MCF7 cells (Figure 3b). Overall these results confirm that caspase-3 activity is essential for HuP10 translocation from the nucleus to the mitochondria.

### Transfer of HuP10 from nucleus to cytoplasm increases the activity of caspase-3

**Figure 5:**
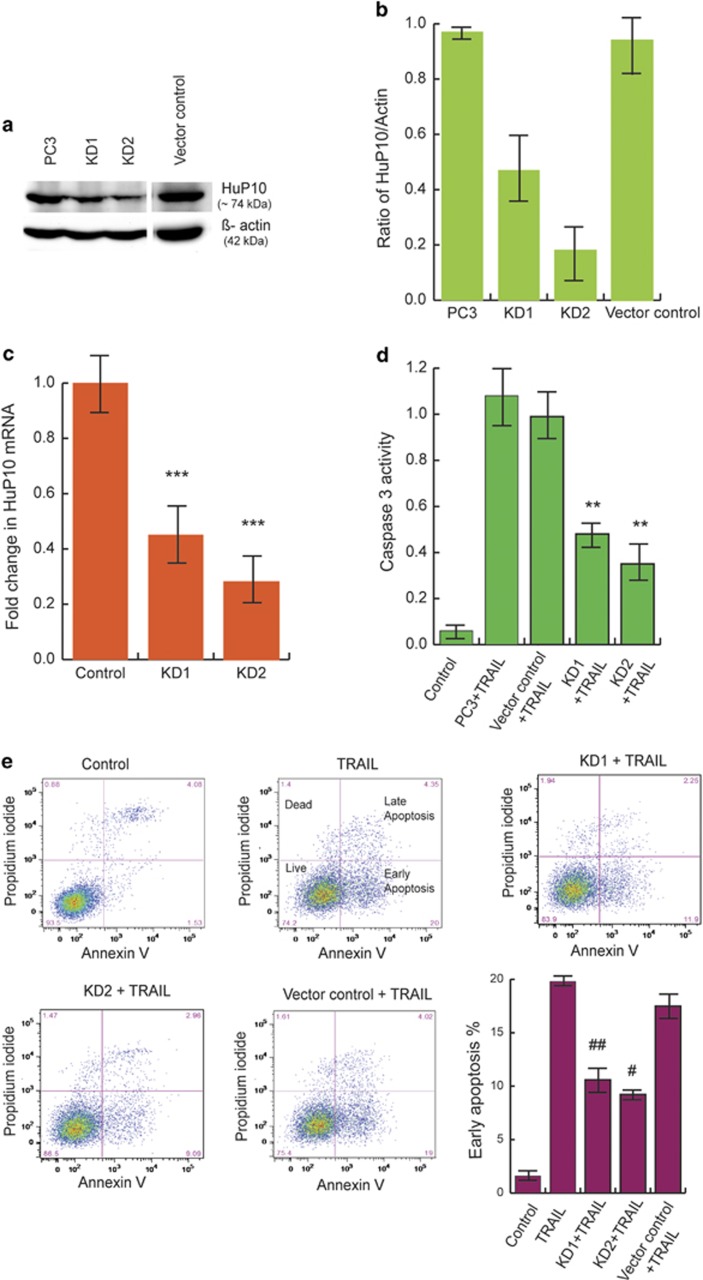
Knockdown of HuP10 reduces TRAIL-induced apoptosis and caspase-3 activity. (**a**) IB analysis of cell lysates of two independent HuP10 KD PC3 clones (KD1 and KD2) showed the reduced amount of HuP10 in the cells. Lysates of normal PC3 and empty vector transfected PC3 cells are used as controls. *β*-actin is the loading control. (**b**) Densitometric analysis of two independent blots as in (**a**), showing ratio of HuP10 to *β*-actin. (**c**) qPCR was used to compare the amount of HuP10 mRNA in PC3 clones KD1 and KD2 with normal PC3 cells as control to confirm the knockdown of HuP10. Results are normalized to HPRT mRNA. Values are mean±S.E. (*n*=4). ***=*P*<0.001 *versus* control. (**d**) Caspase-3 activity was determined after 12 h TRAIL treatment of PC3 cells, KD1 and KD2 clones, and vector control transfected PC3 cells. The control was untreated PC3 cells. Values are mean±S.E. (*n*=3). **=*P*<0.01 *versus* PC3+TRAIL. (**e**) PI/Annexin V analysis of apoptosis in PC3 cells, KD1 and KD2 clones, and vector control transfected PC3 cells after treatment with TRAIL for 12 h. The flow cytometry profile represents Annexin V and Propidium iodide staining along X and Y axis, respectively. The values shown in the four quadrants of each panel are as in Figure 2a. The bar graph shows the early apoptotic cells (%). Values are mean±S.E. (*n*=3). ## and # are *P*=0.01 and *P*=0.001, respectively, *versus* TRAIL

Inhibition of either CRM1 or caspase-3 restricts the TRAIL-induced nuclear export of HuP10 and reduces PARP cleavage (Figures 2 and 3). Therefore, to determine the effect of less of HuP10, we used shRNA to KD HuP10 in PC3 cells. Two selected clones (KD1 and KD2) showed reduced expression of HuP10 as observed by decreased levels of both protein (Figure 5a and b) and mRNA (Figure 5c). Both clones showed a significant reduction of TRAIL-induced caspase-3 activity that correlated to the decreased level of HuP10 (Figure 5d, compare with Figure 5a–c). Furthermore, KD of HuP10 reduced TRAIL-induced apoptosis as measured by Annexin V staining (Figure 5e).

**Figure 6:**
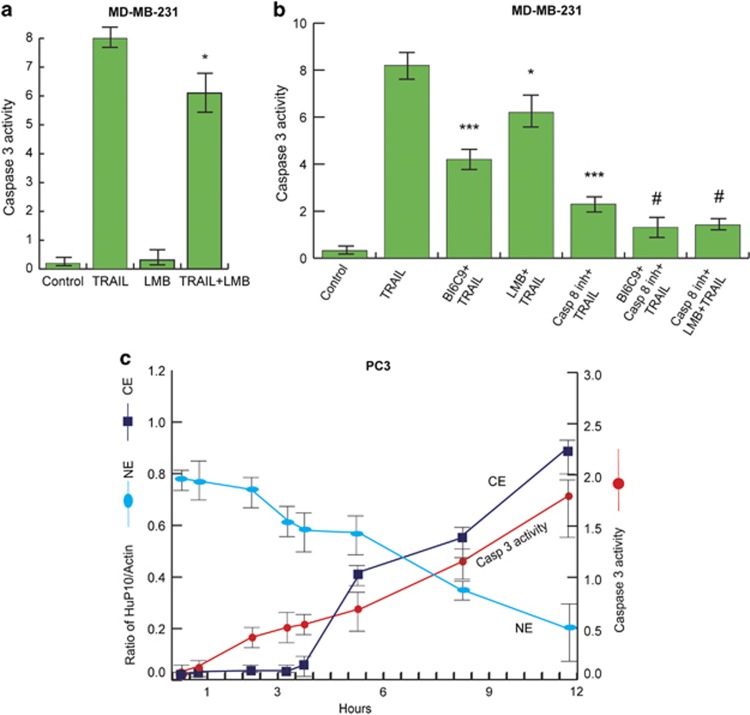
Inhibitors of CRM1, tBID and caspase-8 reduce caspase-3 activity. (**a**) Caspase-3 activity of MDA-MB-231 cells was determined after 2 h LMB (5 ng/ml) followed by 3 h TRAIL (plus LMB) treatment. Values are mean±S.E. (*n*=3). *=*P*<0.05 *versus* TRAIL. (**b**) Caspase-3 activity of MDA-MB-231 was determined after treatment by different agents followed by TRAIL (plus agents) for 3 h. Single treatments were with tBID inhibitor (BI6C9, 100 *μ*M, 24 h), LMB (5 ng/ml, 2 h) or caspase-8 inhibitor (Z-IETD-FMK, 40 *μ*M, 3 h). Double treatments were addition of Z-IETD-FMK after 21 h of BI6C9 treatment (3 h double, 24 h total) and addition of LMB after 1 h of Z-IETD-FMK treatment (2 h double, 3 h total). Values are mean±S.E. (*n*=3). * and *** are *P*<0.05 and *P*<0.001, respectively, *versus* TRAIL. ^**#**^=*P*<0.05 *versus* caspase-8 inhibitor +TRAIL. (**c**) Time course of HuP10 movement and caspase-3 activity. PC3 cells were treated with TRAIL for the time periods as indicated. Then HuP10 levels (relative to *β*-actin) in nuclear (NE) and cytoplasmic (CE) fractions, and caspase-3 activity were determined. Values are mean±S.E. (*n*=2)

Although inhibition of caspase-3 confines most of the HuP10 to the nucleus even after TRAIL treatment (Figure 3a), caspase-3 activity is itself reduced when HuP10 is restricted to the nucleus by LMB (Figure 6a). In the absence of TRAIL treatment, levels of caspase-3 activity in MDA-MB-231 cells are unaffected by LMB (Figure 6a), which is consistent with LMB not affecting cell viability (Supplementary Figure S5B) nor causing PARP cleavage (Figure 2c).

To further understand this reciprocal relationship between caspase-3 activity and HuP10 transport, we treated MDA-MB-231 cells with several different inhibitors either alone or in combination, before TRAIL treatment and then determined caspase-3 activity (Figure 6b). We used Z-IETD-FMK, BI6C9 and LMB as inhibitors of caspase-8, tBID and nuclear export, respectively. As expected, all three inhibitors independently reduced caspase-3 activity, with the caspase-8 inhibitor showing the maximum effect and LMB the least (Figure 6b). This is consistent with caspase-8 acting early, followed by tBID and HuP10 transport. The combination of the tBID inhibitor or LMB with the caspase-8 inhibitor decreased caspase-3 activity more than the caspase-8 inhibitor alone (Figure 6b). The tBID inhibitor and LMB showed about equal effects in these combinations. This suggests that both tBID and translocated HuP10 increase caspase-3 activity independently of caspase-8. Moreover, caspase-3 activity appears to significantly correlate with the movement of HuP10 into the cytoplasm (Figure 6c). Overall, these observations suggest that caspase-3 is not only necessary for translocation of HuP10 out of the nucleus, but that HuP10 also has a role in modulating caspases-3 activity in the cytoplasm.

### HuP10 translocation affects only some apoptosis-related proteins

**Figure 7:**
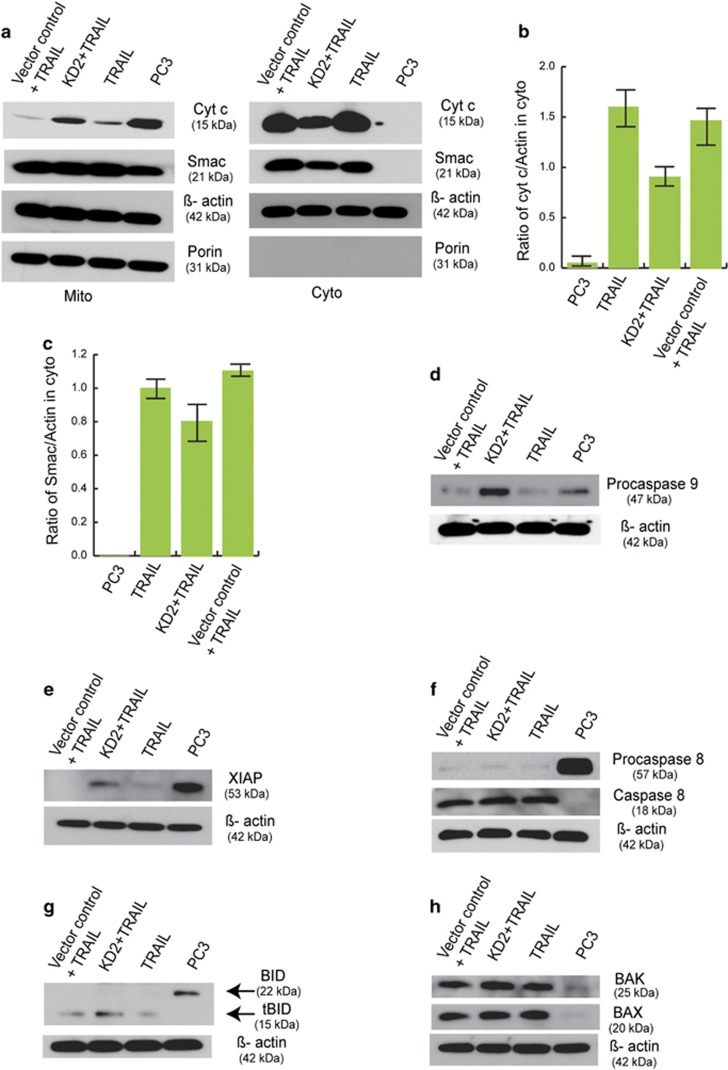
Effects of HuP10 knockdown on apoptosis-related proteins. (**a**) IB analysis of mitochondrial and mitochondria-depleted cytoplasmic fractions of TRAIL-treated and untreated PC3 cells using anti-cytochrome c and anti-SMAC antibodies. There are reduced cytochrome c and SMAC signals in the cytoplasm of TRAIL-treated HuP10 KD cells than TRAIL-treated PC3 cells. Porin is the mitochondrial marker. *β*-actin is the loading control here and for all IB analyses below. (**b**) Densitometric analysis of two independent blots as in (**a**), showing ratio of cyt c to *β*-actin in cytoplasm. (**c**) Densitometric analysis of two independent blots as in (**a**), showing ratio of SMAC to *β*-actin in cytoplasm. (**d**) IB analysis showing more procaspase-9 in TRAIL-treated HuP10 KD cells than TRAIL-treated PC3 cells. (**e**) IB analysis showing more XIAP in TRAIL-treated HuP10 KD cells than TRAIL-treated PC3 cells. (**f**) IB analysis showing no significant difference in caspase-8 in TRAIL-treated PC3 and HuP10 KD cells. (**g**) IB analysis showing no significant difference in tBID in TRAIL-treated PC3 and HuP10 KD cells. (**h**) IB analysis showing no significant difference in BAK and BAX in TRAIL-treated PC3 and HuP10 KD cells

As shown before (Figure 1f and g), *in vivo*, HuP10 moves from the nucleus to the mitochondria after TRAIL treatment, with concurrent release of cytochrome c from the mitochondria. We used a HuP10 KD clone to further confirm HuP10-mediated release of mitochondrial proteins. Less cytochrome c and SMAC is released from the mitochondria into the cytoplasm of the KD clone in response to TRAIL treatment when compared with a scramble shRNA infected control (Figure 7a–c). We also checked the levels of procaspase-9 and XIAP in the HuP10 KD clone, because these proteins are modified after interacting with the cytochrome c and SMAC, respectively, that are released from the mitochondria. In comparison with controls, significant amounts of these proteins are retained in HuP10 KD cells in response to TRAIL (Figure 7d–e). This suggests that in HuP10 KD cells, there is a decrease in the activation of procaspase-9 and in sequestration of XIAP owing to a reduction in the amounts of cytochrome c and SMAC released from the mitochondria.

As shown above, inhibition or KD of caspase-8 had no effect on TRAIL-induced HuP10 movement (Figures 3a and 4g). Neither did TRAIL treatment of a HuP10 KD clone have any effect on caspase-8 or its substrate BID (Figure 7f–g). Both procaspase-8 and BID were nearly completely cleaved after TRAIL treatment of both control and HuP10 KD cells. Similarly, the levels of BAK and BAX remain the same after TRAIL treatment of these cells (Figure 7h).

## Discussion

### HuP10 appears to be a component of the apoptosis pathways

Apoptosis is primarily a cytosolic event.^36, 37^ Signals to initiate apoptosis can come from outside or within the cell. Our study indicates that HuP10, a nuclear protein, moves from nucleus to mitochondria and further activates caspase-3 during apoptosis. This movement of HuP10 requires active caspase-3, but not caspase-8 (Figures 3 and 4 and Supplementary Figure S6). Although caspases-3 and -7 have overlapping activities, inhibition or KD of caspase-3 alone is sufficient to block HuP10 translocation. This is consistent with previous reports that these two caspases are functionally distinct.^33, 38^ HuP10 movement is not directly affected by p53, because it is observed in PC3 cells, which are p53 null.

We observed reduced TRAIL-induced caspase-3 activity in shRNA-mediated HuP10 KD clones of PC3 cells (Figure 5d) similar to a previous study^6^ of siRNA against *PUS10* in HeLa cells. We also show reduction of TRAIL-induced cell death in shRNA-based HuP10 KD PC3 cells (Figure 5e) like that shown in HeLa cells.^39^

### Possible mechanisms for caspase-3-mediated HuP10 movement

Caspase-3 may cause HuP10 translocation directly or indirectly. The predicted molecular weight of HuP10 is ~60 kDa. Recombinant HuP10 isolated from *Escherichia coli* is in this size range (Supplementary Figures S1F–S1G). However, in IBs of all cell lines, it appears to be slightly larger (Supplementary Figure S1G), suggesting that HuP10 in human cells might be modified. Caspase-3 can cleave recombinant HuP10 (Supplementary Figure S1H), as also observed by others.^39^ They also found a ~54 kDa cleaved HuP10 (DOBI) in TRAIL- or etoposide-treated HeLa cells. We did not detect this ~54 kDa HuP10 derivative after TRAIL treatment in any of our cell lines (Supplementary Figure S1D and data not shown). Both our antibody and theirs recognized the caspase-3-cleavage product of recombinant HuP10 (Supplementary Figure S1H). However, if any cleaved HuP10 was present in the cell lysates, our antibody did not recognize it. Therefore, whether caspase-3 can cleave HuP10 itself inside the cell needs further investigation.

The cell lines used in our study are type II,^6, 30, 40, 41, 42^ where via tBID, there is ‘cross-talk’ between the extrinsic and the intrinsic pathways of apoptosis. Therefore, tBID should be activated during TRAIL-induced apoptosis in these cells. We showed this by the reduction of caspase-3 activity using a tBID inhibitor in MDA-MB-231 cells (Figure 6b). However, caspase-3 activity was also reduced by LMB (Figure 6a and b), which inhibits HuP10 movement (Figure 2d) but is not known to affect tBID activity. HeLa cells have limited sensitivity to TRAIL and need to be sensitized. Therefore, we used TRAIL-sensitive PC3 and MDA-MB-231 cells in these studies. When either LMB or a tBID inhibitor was combined with caspase-8 inhibition, the reduction in caspase-3 activity was equivalent (Figure 6b). Furthermore, HuP10 movement to the mitochondria releases cytochrome c and SMAC and this release was reduced in HuP10 KD cells (Figure 7a–c). All these results suggest that translocation of HuP10 from the nucleus to the mitochondria releases mitochondrial contents and activates caspase-3. This resembles the tBID-induced release of mitochondrial contents and activation of caspase-3, but is independent of tBID. Our results could also explain the effects of siRNA against *PUS10* and *BID* mimicking each other.^6^ Procaspase-8 and BID were completely cleaved in both normal PC3 and HuP10 KD PC3 cells (Figure 7f and g), further suggesting no role for caspase-8 and tBID in the HuP10-induced release of mitochondrial contents. BAK and BAX, pro-apoptotic proteins that regulate mitochondrial content release in the intrinsic pathway of apoptosis also appear to have no direct role in the HuP10-induced release of these contents.

Caspase-3 might release HuP10 indirectly by cleaving another protein that prevents its exit from the nucleus. Alternatively, caspase-3 may activate a protein needed for the export of HuP10.

### A proposed feedback loop between HuP10 and caspase-3

The reciprocal relationship between caspase-3 activity and HuP10 translocation is surprising. A downstream feedback amplification loop of caspases during apoptosis has been suggested and mitochondrial contents were proposed as an amplifier in this loop.^1, 13^ After UV irradiation, cytochrome c release from mitochondria is delayed in cells lacking caspase-3 and -7 activity,^38^ suggesting an effect of downstream caspases on upstream events. This may be owing to the lack of caspase-3, preventing HuP10 translocation to the mitochondria and delayed cytochrome c release. We believe that caspase-3-mediated translocation of HuP10 from the nucleus to the mitochondria causes the reciprocal amplification of caspase-3, because as the HuP10 moves to the cytoplasm, the activity of caspase-3 increases (see Figure 6c). Initiator caspases presumably cause the initial activation of caspase-3 whereas the later amplification of caspase-3 activity is effected by the translocation of HuP10.

**Figure 8:**
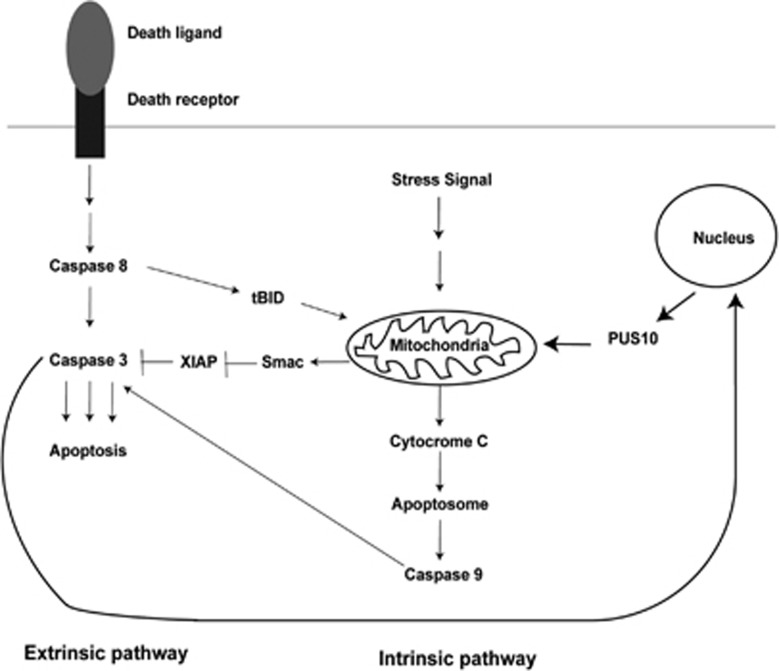
A general model for the role of PUS10 in apoptosis. Our study revealed caspase-3-mediated translocation of PUS10 from nucleus to mitochondria (thick lines) with concurrent release of cytochrome c and SMAC, and a reciprocal increase in caspase-3 activity. Release of cytochrome c could increase caspase-3 activity through the apoptosome and caspase-9 pathway and SMAC released from the mitochondria could sequester XIAP, thus removing inhibition of caspase-3 activity

We propose a model for the apoptosis that includes this feedback amplification loop (Figure 8). In this model caspase-3 causes translocation of PUS10 from the nucleus to the mitochondria and this releases cytochrome c, SMAC and so on. The amplification of caspase-3 activity could occur by cytochrome c forming the apoptosome and activating caspase-9. In addition, this amplification might occur by sequestration of XIAP by SMAC, thus removing XIAP inhibition of caspase-3 (and caspases-7 and -9). It has been shown that SMAC release from mitochondria is caspase-dependent^43^ and that SMAC-induced apoptosis is caspase-3-dependendent.^44^ Removal of XIAP inhibition of caspase-3 would allow (a second) auto-cleavage of caspase-3 to remove its pro-domain and make it fully active. This XIAP sequestration mechanism has been suggested as an amplifier of caspase-3 activity.^1, 45^ The precursor of caspase-3 is a 36 kDa (p36) protein. Initiator caspases cleave it to a partially active enzyme consisting of p24 and p12 subunits.^45, 46^ Auto-cleavage of p24 converts it to a p20/p17 subunit, which along with the p12 subunit forms the fully active caspase-3. XIAP interacts with p24 preventing its auto-cleavage.^45^ Such interactions can explain the reciprocal relationship between caspase-3 activity and HuP10 movement from the nucleus to the mitochondria. This relationship can also explain the effects of caspase cleavage in a previous siRNA study.^6^ In HeLa cells (type II cells), siRNA against either *BID* or *PUS10* alone did not affect caspase-3 cleavage, but siRNA against the caspase-8 gene did block caspase-3 cleavage. This suggests that both the BID- and PUS10-mediated pathways redundantly activate caspase-3, presumably through the release of mitochondrial contents. Furthermore, the caspase-8 activity needed to cleave procaspase-3 during TRAIL-induced apoptosis is upstream of both the BID- and PUS10-mediated pathways. It is likely that HuP10 enhances caspase-3 activity by both the cytochrome c-mediated activation of caspase-9 and by the auto-cleavage of pro-domain of caspase-3 mediated by the XIAP/SMAC interaction. SMAC inactivation of XIAP also activates caspase-9.

The loop-like relationship between caspase-3 and PUS10, comprising caspase-3-mediated HuP10 translocation from nucleus to mitochondria, cytochrome c and SMAC release from mitochondria, followed by activation of caspase-9, as well as sequestration of IAPs to further activate caspase-3, would be common to all pathways of apoptosis that involve caspase-3. This would not occur in cells that lack functional caspase-3, such as MCF7. Our preliminary results indicate that the treatment of cells with several apoptotic agents, for example, etoposide, staurosporine, ursolic acid, H_2_O_2_ and so on, also causes movement of HuP10 (S Jana, M Deogharia, M Bosmeny and R Gupta, unpublished).

In summary, we have shown that PUS10, a nuclear protein, moves to mitochondria, releasing cytochrome c and SMAC during TRAIL-induced apoptosis. Caspase-3 is required for this CRM1-mediated nuclear export of PUS10 and translocated PUS10 reciprocally activates caspase-3, creating an amplification loop. Therefore, any factor that interferes with the movement of HuP10 or with its interaction with the mitochondria would reduce the sensitivity of tumor cells to TRAIL. These findings may be useful in developing drugs for cancer therapy and for understanding the side effects of cancer drugs, such as SMAC mimetics.

## Materials and methods

### Cell cultures

PC3 and LNCaP cells were cultured in RPMI 1640 (ATCC, Manassas, VA, USA), and HeLa, RH30, MDA-MB-231 and MCF7 cells were grown in DMEM media (Hyclone, GE Healthcare Life Science, Logan, UT, USA), both with 10% FBS, 100 U/ml penicillin and 100 *μ*g/ml streptomycin (15140122, Gibco, ThermoFisher, Waltham, MA, USA) under standard culture conditions (37 °C and 5% CO_2_). PC3 cells were purchased from ATCC (CRL-1435) in March 2016. MDA-MB-231 and MCF7 cells were received from Sophia Ran, and LNCaP, HeLa and RH30 cells were received from Kanako Hayashi, Farid Kadyrov and Judy Davie, respectively. RH30 and MDA-MB-231 lines were authenticated in September 2011 (by Biosynthesis, Lewisville, TX, USA) and February 2013 (by ATCC), respectively. All treatments were done at 80–90% confluency. Concentrations of different agents and times of treatment are mentioned in figure legends. Most cells were treated with TRAIL for 12 h. MDA-MB-231 cells were treated for 3 h, because the cells have a high proliferation rate and respond to TRAIL within this period. For sensitization studies, cells were co-treated with CHX and TRAIL (PHC1634, Invitrogen, ThermoFisher, Waltham, MA, USA) for 12 h. For combination treatments, cells were pre-treated with either LMB or inhibitors of tBID (BI6C9, B0185, Sigma, St. Louis, MO, USA) and caspases before addition of TRAIL. The pan-caspase inhibitor (Z-VAD-FMK, ALX-260-020-M001) was from Enzo Life Science, Farmingdale, NY, USA and other caspase inhibitors were from R&D System, Minneapolis, MN, USA. These inhibitors were for caspase-3 (Z-DEVD-FMK, FMK004), -6 (Z-VEID-FMK, FMK006), -8 (Z-IETD-FMK, FMK007), -9 (Z-LEHD-FMK, FMK008) and -10 (Z-AEVD-FMK, FMK009).

### Immunofluorescence studies

Cells were grown on coverslips placed in 60-mm plates and experiments were performed after about 80% confluency. MitoTracker Red (CMXRos, M7512, Invitrogen) was added into the culture media (without FBS) at final concentrations of 300 nM and cells were incubated for 15 min (MDA-MB-231 cells) or 1 h (other cells) at 37 °C (with 5% CO_2_). The culture medium was removed. All further treatments were done at room temperature in small volumes, just enough to cover the coverslip. The cells were fixed in 4% paraformaldehyde in PBS (19943, Affymetrix USB, Santa Clara, CA, USA) for 15 min and then washed with PBS (137 mM NaCl, 2.7 mM KCl, 10 mM Na_2_HPO_4_, 1.8 mM KH_2_PO_4_). The cells were then treated with 10% Normal Goat Serum (NGS, 0060-01, Southern Biotech, Birmingham, AL, USA) (for single IF) or 1% BSA (for double IF) in PBS containing 1% NP40 for 1 h and then washed with PBS. Primary antibody(ies) diluted (1:100) in PBS containing either 10% NGS or 1% BSA was added and incubated for 2 h. After the PBS wash, cells were incubated in secondary antibody(ies) diluted 0.3 *μ*l in 100 *μ*l of PBS containing either NGS or BSA for 1 h. Primary antibodies were rabbit anti-human Pus10 (HPA049582, Sigma) and anti-cytochrome c (ab90529, Abcam, Cambridge, MA, USA), and mouse anti-tubulin (A11126, Life Technologies, ThermoFisher, Waltham, MA, USA). Secondary antibodies were Alexa Fluor 488 (green) goat anti-rabbit IgG (A11034, Invitrogen), Alexa Fluor 568 (red) goat anti-rabbit IgG (A11011, Invitrogen) and Alexa Fluor 350 (blue) donkey anti-mouse IgG (A10035, Invitrogen). The cells were then washed with PBS and incubated in 1 *μ*M DAPI for 5 min to stain the nuclei. Finally, the cells were washed four times with PBS and the coverslips mounted face down onto glass slides using immersion oil (M3004, ThermoFisher). Slides were viewed under Type F immersion liquid (11513859, Leica Biosystems, Buffalo Grove, IL, USA) using a Leica DMi8 microscope or Type HF immersion oil (16245, Cargille Laboratories, Cedar Grove, NJ, USA) using a Nikon Eclipse E800 microscope. As caspase-3 and caspase-8 shRNA constructs contain GFP markers (green), we used the Alexa Fluor 568 (red) secondary antibody for IF analyses of KD of these caspases, instead of the Alexa Fluor 488 (green) as used for all other IF analyses.

### Preparation of cellular fractions and cell lysate

Nuclear and cytoplasmic fractions were prepared by slight modification of a published procedure.^47^ A trypsinized and harvested cell pellet was re-suspended in PBS containing 0.1% NP40 and protease inhibitor (88665, Pierce ThermoFisher, Waltham, MA, USA, 1 mini tablet/10 ml) and centrifuged at 900 × *g* for 5 min at 4 °C. The supernatant was saved as the cytoplasmic fraction. The pellet was washed once with PBS containing 0.1% NP40 and protease inhibitor, and again centrifuged at 900 × *g* for 5 min at 4 °C. The pellet was re-suspended in PBS containing 0.1% NP40 and protease inhibitor, and sonicated three times for 3–5 s at 3 watts each, to prepare the nuclear fraction. The fractions were stored at −80 °C. Mitochondrial and mitochondria-depleted cytoplasmic fractions were prepared by using a Cytosol/Mitochondria Fractionation kit (ab65320, Abcam).

To prepare total cell lysate, cells were washed with PBS, trypsinized and harvested by centrifugation at 200 × *g* for 2 min at 4 °C. The pellet was re-suspended in RIPA buffer (50 mM Tris-Cl, pH 8.0, 150 mM NaCl, 0.1% SDS, 0.5% deoxycholate, 1% NP40, 1 mM EDTA, 1 mM DTT) containing protease inhibitor and incubated on ice for 10 min. The cell suspension was then vortexed for 10 s and kept on ice for another 10 min. This was done thrice. The lysate was then centrifuged at 16 000 × *g* for 10 min at 4 °C and the clear supernatant was saved as the cell lysate. Protein concentrations of the fractions were determined by a Coomassie protein assay kit (23200, Pierce).

### Immunoblotting

Appropriate fractions (~40 *μ*g protein/lane) were separated by SDS-polyacrylamide gel electrophoresis (10 or 15%) and electroblotted onto PVDF membranes. Blots were then blocked with 2% (w/v) BSA in TBST buffer (10 mM Tris-Cl, pH 7.6, 100 mM NaCl and 0.1% Tween-20) for 1 h at room temperature. The primary antibodies (diluted in TBST according to manufacturer's recommendation) were added and shaken overnight at 4 °C, followed by 4 h incubation with 1 : 10 000 dilution of anti-rabbit IgG HRP-linked secondary antibody (NA934V, GE Healthcare) in TBST at room temperature. Chemiluminescence was detected using Super Signal West Pico Chemiluminescence Substrate kit (34078, ThermoFisher). The signal was captured using a UVP ChemiDoc-ItTS2 imaging system (UVP, Upland, CA, USA). Densitometric analysis of the image was done using ImageJ 1.46r. The human Pus10 and cytochrome c antibodies used for IF study were also used for IB. Other primary antibodies were rabbit antibodies against PARP (9542, Cell Signaling, Danvers, MA, USA), BAK (12105, Cell Signaling), BAX (2772, Cell Signaling), SMAC (2954, Cell Signaling), Caspase-3 (9662, Cell Signaling), Procaspase-8 (4790, Cell Signaling), Caspase-8 (9496, Cell Signaling), Procaspase-9 (9502, Cell Signaling), BID (2002, Cell Signaling), XIAP (610762, BD Biosciences, San Jose, CA, USA), lamin A (ab26300, Abcam), tubulin (ab6046, Abcam) and *β*-actin (ab8227, Abcam).

### Determination of the specificity of anti-HuP10 antibody

The immunogen for the commercial polyclonal rabbit antibody against HuP10 used here covers amino acids N270 to E375 (see sequence of HuP10 in Supplementary Figure S1A). This antibody recognizes HuP10 in IF of cells and IB of cell lysates and cellular fractions as well as recombinant HuP10 (Supplementary Figures S1C-S1H). This antibody also recognizes an ~48 kDa unknown protein (Supplementary Figures S1D–S1E). The corresponding antigen (APREST83448, Sigma) is not available commercially. Therefore, we used recombinant HuP10 to test the specificity of the commercial antibody in our IF studies. For this we used the above described IF procedure except we added 2 *μ*g of recombinant HuP10 to diluted primary antibody solution and incubated it for 1 h at 4 °C before adding the antibody to the cells (Supplementary Figure S1I).

### Cell viability assay

Vybrant MTT Cell Proliferation Assay Kit (V13154, Invitrogen) was used for the cell viability assays according to the manufacturer's instructions. Cells were plated in 96-well plates at a density of 1–1.2 × 10^4^ cells/well, grown for 24 h and followed by incubation with appropriate reagent. Absorbance was recorded at 540 nm wavelengths using BioTek SynergyMx micro plate reader (BioTek, Winooski, VT, USA). Each treatment was replicated in three different wells and independently performed three times. Cell viability was determined as (100−death rate). Death rate was calculated by the formula: absorbance of [(control−treated)/control] × 100 according to a published procedure.^48^

### Annexin V analysis

After TRAIL, LMB or LMB+TRAIL treatment, cells were washed with PBS and suspended in 1 × BD binding buffer (51-66121E, BD Sciences). Next, Propidium iodide (P4864, Sigma) and Annexin V (550474, BD Sciences) were used to stain the cells and incubated for 15 min at RT in the dark. The labeled cells were detected by a BD FACS Canto flow cytometer. At least 20 000 events were recorded per sample condition and analyzed by FlowJo software.

### Caspase-3 activity assay

Caspase-3 activity was determined using a colorimetric caspase-3 assay kit (ab39401, Abcam) according to the manufacturer's protocol. Cell lysate containing 200 *μ*g (MDA-MB-231 cells) or 400 *μ*g (PC3 cells) protein was used for each assay. Each assay was repeated with three independent cell cultures. Absorbance at 400 nm was recorded using a BioTek SynergyMx micro plate reader. Caspase activity was calculated in arbitrary units using the formula: absorbance of (sample−control)/control.

### Preparation of recombinant human Pus10

The HuP10 cDNA clone (8069212) was purchased from Open Biosystems, Dharmacon, Lafayette, CO, USA. It was used as a template to amplify the gene by PCR using a forward (5′-CGCGGATCCATGTTCCCACTGACTGAGGAAAACAAGC-3′) and a reverse (5′-CGCCTCGAGTTACTAGTCATCCAGAGCAGGTGG-3′) primer. The HuP10 gene was cloned between the *Bam*HI and *Xho*I sites of pET28a (Novagen, EMD Millipore, Danvers, MA, USA) vector. The expressed recombinant protein contains an N-terminal 6xHis tag. Proteins were induced at 0.6 OD_600_ using 0.8 mM IPTG at 37 °C for 2 h in *E. coli* Rosetta (DE3) pLysS cells. Induced pellets were re-suspended in lysis buffer (20 mM Tris-Cl, pH 7.5 and 150 mM NaCl) containing PMSF (1 mM) and lysozyme (1 mg/ml) and incubated at 37 °C for 30 min, followed by another incubation on ice for 1 h. Cells were lysed by sonication and centrifuged. Cleared supernatant was loaded onto a Ni-NTA column equilibrated in the same buffer. The protein was eluted in the same buffer containing 300 mM imidazole. Eluted protein was dialyzed in buffer (20 mM Tris-Cl, pH 7.5, 150 mM NaCl, 3 mM MgCl_2_, 0.2 mM EDTA and 20% glycerol) overnight at 4 °C. Thrombin (1 mM) was used to cut the His tag from purified recombinant HuP10.

### Caspase-3 cleavage of recombinant HuP10

Recombinant HuP10 (2 *μ*g) was incubated with 100 units of caspase-3 (235417, Calbiochem, San Diego, CA, USA) in a total volume of 20 *μ*l containing buffer (100 mM, 50 mM Hepes, 10 mM DTT, 1 mM EDTA, 0.5% CHAPS and 10% glycerol, pH 7.4) for 2.5 h at 30 °C. The reaction was immunoblotted using anti-HuP10 antibody.

### Preparation of KD clones

To prepare HuP10 KD clones, ~0.4 × 10^5^ cells were seeded in 12-well plates with 2 ml RPMI media for 24 h. Transfection was done using TurboFect (R0531, ThermoFisher) using 2 *μ*g shRNA (TRCN0000133677 and TRCN0000134366, ThermoFisher) or control plasmid (pLKO.1, RHS4080, ThermoFisher). Puromycin (3 *μ*g/ml) was added for selection. Media was changed every 3–4 days with fresh antibiotic containing media. Single colonies were picked after 3–4 weeks.

pGIPZ mir30-based lentiviral vectors containing the sense sequences 5′-CCGACAAGCTTGAATTTAT-3′ and 5′-GACAAAGTTTACCAAATGA-3′ (for caspase-3), and 5′-GACAAAGTTTACCAAATGA-3′ and GGGTCGATCATCTATTAAT-3′ (for caspase-8) were used to KD the expression of the two caspases in PC3 cells. 293 T cells were transfected with lentiviral vector plasmids and packaging plasmids psPAX2 and pMD2.G. Supernatants containing lentiviruses were collected on the second day after transfection and concentrated by centrifuging at 5000 rpm for 24 h at 4 °C. For lentiviral transduction, PC3 cells were seeded at 70–80% confluency on six-well plates. Cells were pre-treated with polybrene (8 *μ*g/ml) and infected with concentrated lentivirus. Puromycin (3 *μ*g/ml) was added for selection of KD clones.

### qPCR to determine KD of mRNA

Total RNA was isolated using TRI Reagent (TR118, Molecular Research Center, Cincinnati, OH, USA), followed by DNase I (M0303S, New England Biolabs, Ipswich, MA, USA) digestion and cDNA was prepared using MultiScribe MuLV reverse transcriptase and random primer mix (4368814, Applied Biosystem, ThermoFisher, Waltham, MA, USA). The SYBER green PCR mix (170-8880, BioRad, Hercules, CA, USA) and primers (forward (5′-GAGGATTGCCACTTCCTAGC-3′) and reverse (5′-CAAGAACAGCGCATACAGCC-3′) for HuP10, forward (5′-TCTGGTTTTCGGTGGGTGTG-3′) and reverse (5′-CGCTTCCATGTATGATCTTTGGTT-3′) for caspase-3, forward (5′-CTGCTGGGGATGGCCACTGTG-3′) and reverse (5′-TCGCCTCGAGGACATCGCTCTC-3′) for caspase-8) were used for qPCR. Relative levels of gene expression were normalized against standard HPRT (for HuP10) and Actin (for caspase-3 and caspase-8) genes. All quantitative PCR assays were performed in triplicate from four independent RNA samples.

### Statistical analysis

Cell viability and caspase-3 activity data are representative of three independent experiments performed in triplicate and results are expressed as mean±S.E. The *P-*values were determined by Student's *t*-test using IBM SPSS software version 24.

## Publisher’s note:

Springer Nature remains neutral with regard to jurisdictional claims in published maps and institutional affiliations.
