# Supplementary material for: Reciprocal amplification of caspase-3 activity by nuclear export of a putative human RNA-modifying protein, PUS10 during TRAIL-induced apoptosis
Source: Cell Death Dis. 2017 Oct 5;8(10):e3093–. doi: 10.1038/cddis.2017.476 (PMC5682645; doi:10.1038/cddis.2017.476)
Supplement: Supplementary Figures [file cddis2017476x1.pdf]

## SUPPLEMENTARY FIGURE LEGENDS

**Supplementary Figure S1. Amino acid sequence of human PUS10 and recognition of the protein by the commercial antibody used in this study.**

(A) Amino acid sequence of HuP10 protein. Predicted Nuclear Localization Signal (NLS) sequence is shown in red. Immunogen for the commercial anti-human PUS10 antibody used in this study covers the region N270-E375.

(B) Multiple sequence alignment of the NLS containing region of PUS10 of some mammals: mouse (*Mus*), bat (*Miniopterus*), human (*Homo*), cow (*Bos*) and goat (*Capra*).

(C) IB analysis of cell lysates using anti-HuP10 antibody showed that HuP10 is present in all six cell lines used in this study. ß-actin was used as a loading control. Positions of two size markers are indicated on the side.

(D) Size of HuP10 remains about the same after TRAIL treatment. IB analysis of cell lysates of PC3 cells treated with TRAIL for 24 h using anti-HuP10 antibody. Note: an unknown protein of about 48 kDa is also detected by the antibody.

(E) HuP10 is present in the nucleus but not in the cytoplasm. IB analysis of nuclear (NE) and cytoplasmic (CE) fractions of PC3 cells using anti-HuP10 antibody. Note: the unknown protein observed in (D) is present in both nucleus and cytoplasm.

(F) The anti-HuP10 antibody recognizes recombinant His-tagged HuP10. IB analysis of cell lysates of un-induced and induced *E. coli* cells containing recombinant HuP10 and Ni-affinity purified recombinant HuP10 using anti-HuP10 antibody.

(G) HuP10 present in human cells is larger than recombinant HuP10 (~60 kDa) purified from *E. coli* cells. IB analysis of cell lysate of PC3 cells and purified recombinant HuP10 using anti-HuP10 antibody. Positions of two size markers are indicated on the side.

(H) Caspase-3 can cleave recombinant HuP10. IB analysis of recombinant HuP10 mock-treated (- Casp 3) and treated with caspase-3 (+ Casp 3) for 2.5 h, using anti-HuP10 antibody.

(I) Determination of the specificity of anti-HuP10 for IF studies. Anti-HuP10 incubated with recombinant HuP10 was used for an immunofluorescence study similar to the one in Figure 1A. Untreated antibody was used as control. Mito Tracker and DAPI staining are also as in Fig.1A. Arrows indicate the faint outlines of the cells. Bar = 10 μm.

**Supplementary Figure S2. TRAIL-induced HuP10 movement in some TRAIL sensitive cell lines**

IF analyses of MDA-MB-231 cells treated with TRAIL (0.5 μg/ml) for 3 h (A) and RH30 cells treated with TRAIL for 12 h (B), as shown for PC3 cells in Fig. 1A. Staining and arrows are as in Fig. 1A. Bars = 10 μm.

IB analyses showing PARP cleavage in TRAIL treated MDA-MB-231 (C) and RH30 cells (D) are similar to Fig. 1D, using tubulin as a loading control. See also Supplementary Figs. S4C and S4D.

**Supplementary Figure S3. TRAIL-induced HuP10 movement in cell lines sensitized to TRAIL by cycloheximide**

IB analyses showed greater PARP cleavage after TRAIL+CHX treatment than after TRAIL alone. HeLa (A) and LNCaP (D) cells were treated with CHX (HeLa - 0.5 μM, LNCaP - 1 μM)

and TRAIL (0.5 μg/ml) together or alone for 12 h. Untreated cells are the controls. Tubulin is the loading control. See also Supplementary Fig. S4E and S4F.

The MTT assay showed a significant reduction in cell viability when HeLa (B) and LNCaP (E) cells were exposed to CHX plus TRAIL together. Values are mean ± SE (n=3). In (B) ***=p<0.001 vs untreated control and TRAIL alone, and $^{\#}$=p0.001 vs CHX alone. In (E) ***=p<0.001 vs untreated control, and $^{\#}$=p0.001 vs TRAIL and CHX alone.

IF analyses of HeLa (C) and LNCaP (F) cells after simultaneous treatment with CHX and TRAIL. Staining and arrows as in Fig. 1A. Bars = 10 μm.

**Supplementary Figure S4. Determination of the amount of cleaved PARP under different conditions.**

Densitometric analyses of immunoblots of PARP cleavage showing the ratio of cleaved PARP to ß actin or tubulin. A through H are analyses of Figs. 1D, 2C, S2C, S2D, S3A, S3D, S6B and S7B, respectively (indicated below each panel). Each analysis is based on at least two independent blots. Error bars indicate mean ± SE.

**Supplementary Figure S5. Determination of leptomycin B sensitivity of PC3 and MDA-MB-231 cells.**

PC3 and MDA-MB-231 cells were treated with different concentrations of LMB for 12 and 5 h, respectively. Cell viability was then determined by MTT assays in three independent experiments with three replicates each. ** and *** are p<0.01 and p<0.001, respectively, vs untreated (0 ng/ml).

**Supplementary Figure S6. Effects of caspase inhibitors on TRAIL-induced HuP10 translocation and apoptosis.**

(A) IF analyses of PC3 cells growing on coverslips at 80% confluency treated simultaneously with inhibitors of two initiator caspases (-8 and -9, -8 and -10, or -9 and -10), 40 µM each for 3 h followed by 12 h TRAIL (0.5 µg/ml, plus inhibitors) treatment. Staining as in Fig. 1A. These inhibitor treatments do not block the transfer of HuP10 to the cytoplasm (arrows). Bars = 10 µm.

(B) IB analyses showing PARP cleavage in MDA-MB-231 cells pre-treated with different caspase inhibitors (concentrations as in Fig. 3A) for 2 h followed by 1 h TRAIL (0.5 µg/ml) plus inhibitors. See also Supplementary Fig. S4G.

(C) Caspase-3 activity of MDA-MB-231 cells was determined after caspase inhibitors and TRAIL treatment as in (B). Values are mean ± SE (n=2).

(D) IB analysis of nuclear (NE) and cytoplasmic (CE) fractions of PC3 cells treated with caspase-3 inhibitor (40 µM) for 3 h followed by 12 h TRAIL (0.5 µg/ml, plus inhibitors) treatment using anti-HuP10 antibody. Lamin A and tubulin are markers to check the purity of NE and CE, respectively. ß-actin was used as a loading control.

**Supplementary Figure S7. HuP10 does not move out of the nucleus during TRAIL-induced apoptosis of sensitized MCF7 cells.**

(A) The MTT assay shows significant difference in MCF7 cell viability after 12 h simultaneous exposure to TRAIL (0.5 µg/ml) and CHX (0.5 µM) when compared to untreated (control) or treatment with TRAIL or CHX alone. Values are presented as mean ± SE (n=3). ***=p <0.001 individually vs control or TRAIL or CHX treatment.

(B) IB analysis of the lysates of the MCF7 cells treated individually with TRAIL and CHX alone or combined for 6 h and 12 h using anti-PARP antibody to determine PARP cleavage. ß-actin was used as a loading control. See also Fig. S4H.

(C) Nuclear (NE) and cytoplasmic (CE) fractions of untreated and TRAIL+CHX-treated (12 h) MCF7 cells were analyzed by IB using anti-HuP10 antibody. Nuclear and cytoplasmic markers were lamin A and tubulin, respectively. ß actin was used as a loading control.

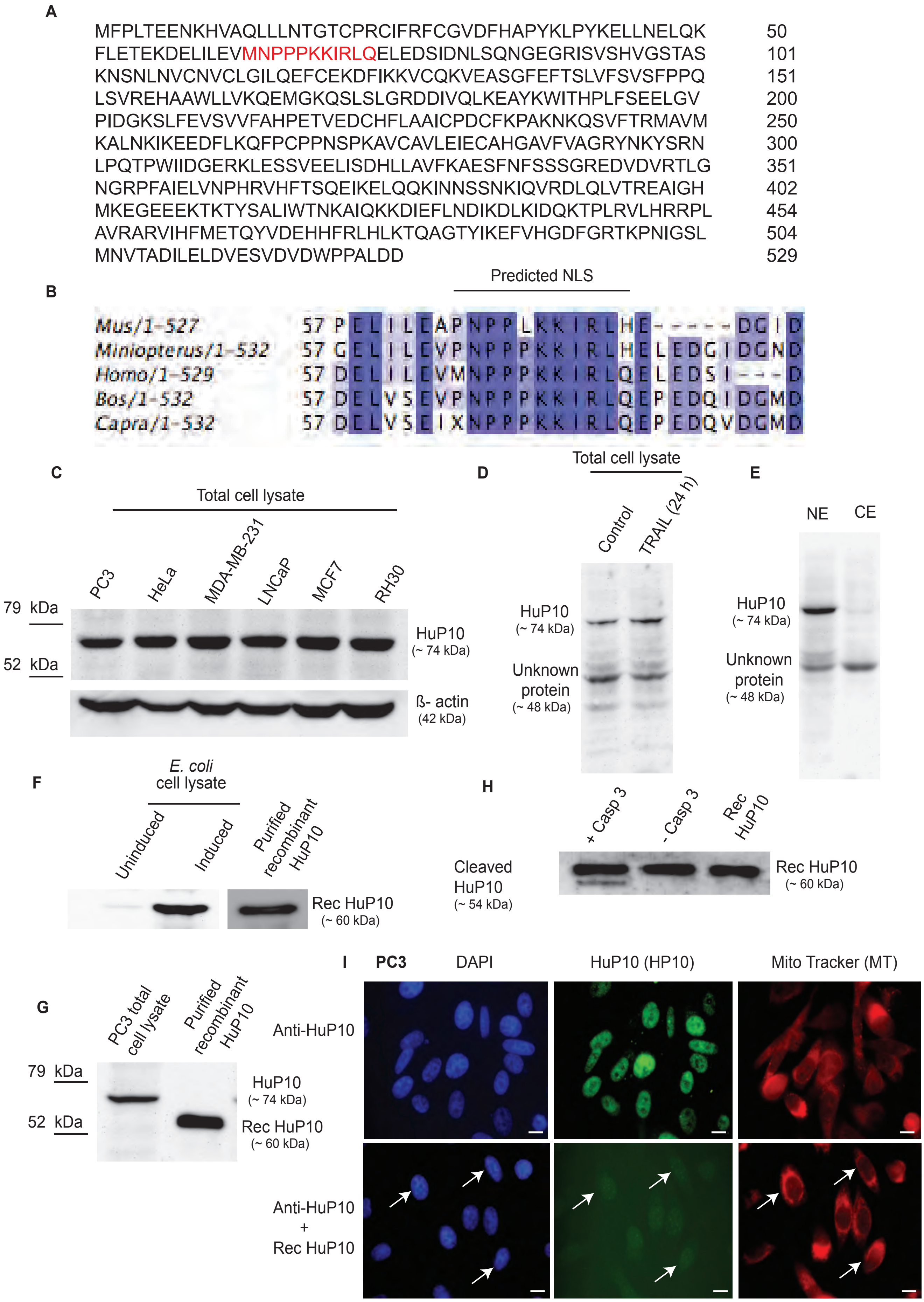
A
MFPLTEENKHVAQLLLNTGTCPRCIFRFCGVDFHAPYKLPYKELLNELQK 50
FLETEKDELILEVMNPPPKKIRLQELEDSIDNLSQNGEGRISVSHVGSTAS 101
KNSNLNVCNVCLGILQEFCEKDFIKKVCQKVEASGFEFTSLVFSVSFPPQ 151
LSVREHAAWLLVKQEMGKQSLSLGRDDIVQLKEAYKWITHPLFSEELGV 200
PIDGKSLFEVSVVFAHPETVEDCHFLAAICPDCFKPAKNKQSVFTRMAVM 250
KALNKIKEEDFLKQFPCPPNSPKAVCAVLEIECAHGAVFVAGRYNKYSRN 300
LPQTPWIIDGERKLESSVEELISDHLLAVFKAESFNFSSSGREDVDVRTLG 351
NGRPFAIELVNPHRVHFTSQEIKELQQKINNSSNKIQVRDLQLVTREAIGH 402
MKEGEEEKTKTYSALIWTNKAIQKKDIEFLNDIKDLKIDQKTPLRVLHRRPL 454
AVRARVIHFMETQYVDEHHFRLHLKTQAGTYIKEFVHGDFGRTKPNIGSL 504
MNVTADILELDVESVDVDWPPALDD 529
B
Predicted NLS
Mus/1-527 57 PELILEAPNPPLKKIRLHE-----DGID
Miniopterus/1-532 57 GELILEVPNPPPKKIRLHELEDGIDGND
Homo/1-529 57 DELILEVMNPPPKKIRLQELEDSI---D
Bos/1-532 57 DELVSEVPNPPPKKIRLQEPEDQIDGMD
Capra/1-532 57 DELVSEIXNPPPKKIRLQEPEDQVDGMD
C
Total cell lysate
PC3
HeLa
MDA-MB-231
LNCaP
MCF7
RH30
79 kDa
52 kDa
HuP10
(~ 74 kDa)
ß- actin
(42 kDa)
D
Total cell lysate
Control
TRAIL (24 h)
HuP10
(~ 74 kDa)
Unknown
protein
(~ 48 kDa)
E
NE
CE
HuP10
(~ 74 kDa)
Unknown
protein
(~ 48 kDa)
F
E. coli
cell lysate
Uninduced
Induced
Purified
recombinant
HuP10
Rec HuP10
(~ 60 kDa)
H
+ Casp 3
- Casp 3
Rec
HuP10
Cleaved
HuP10
(~ 54 kDa)
Rec HuP10
(~ 60 kDa)
G
PC3 total
cell lysate
Purified
recombinant
HuP10
79 kDa
52 kDa
HuP10
(~ 74 kDa)
Rec HuP10
(~ 60 kDa)
I
PC3
DAPI
HuP10 (HP10)
Mito Tracker (MT)
Anti-HuP10
Anti-HuP10
+
Rec HuP10

**Figure S1**

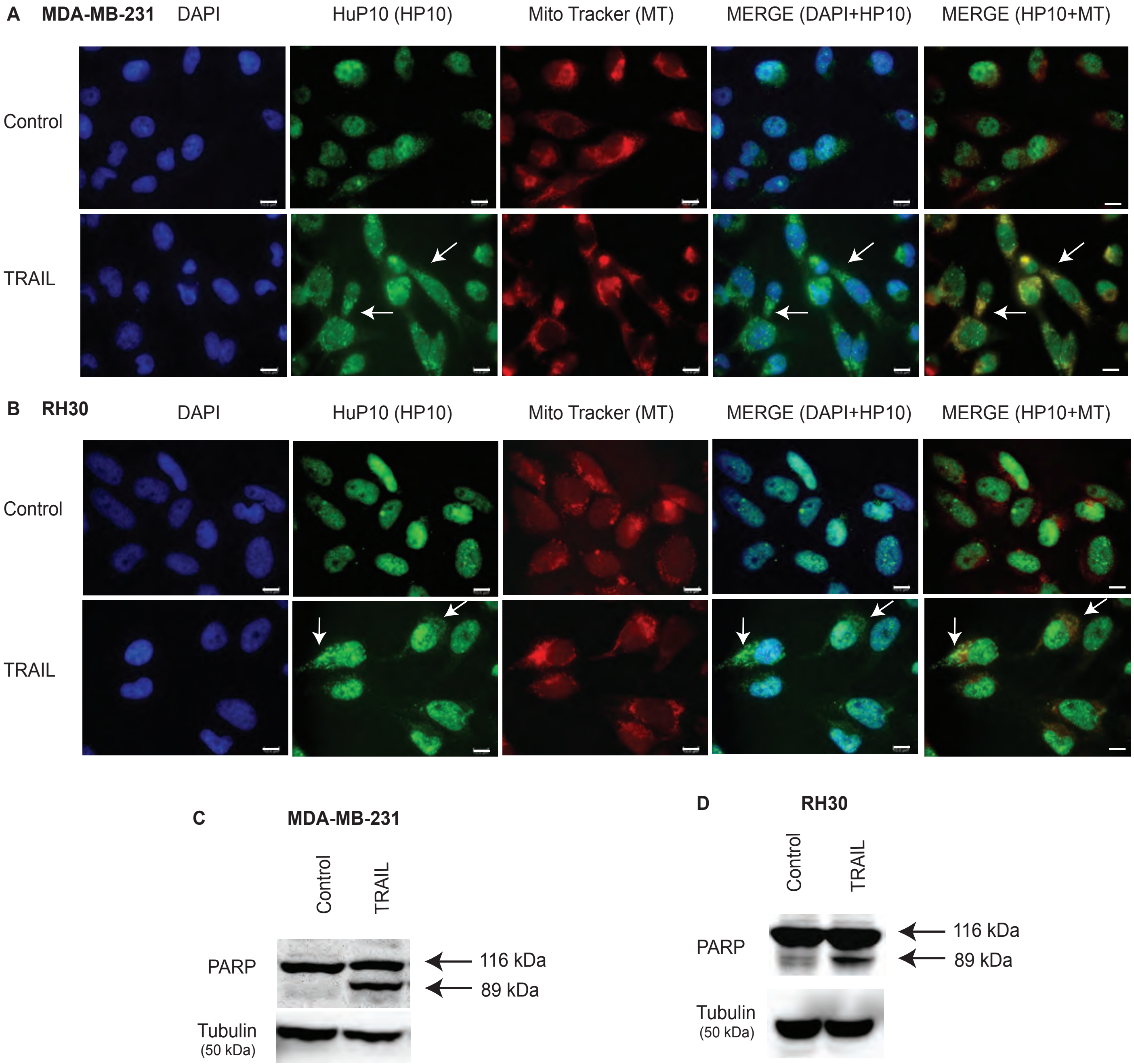
A
MDA-MB-231
DAPI
HuP10 (HP10)
Mito Tracker (MT)
MERGE (DAPI+HP10)
MERGE (HP10+MT)
Control
TRAIL
B
RH30
DAPI
HuP10 (HP10)
Mito Tracker (MT)
MERGE (DAPI+HP10)
MERGE (HP10+MT)
Control
TRAIL
C
MDA-MB-231
Control
TRAIL
PARP
116 kDa
89 kDa
Tubulin
(50 kDa)
D
RH30
Control
TRAIL
PARP
116 kDa
89 kDa
Tubulin
(50 kDa)

**Figure S2**

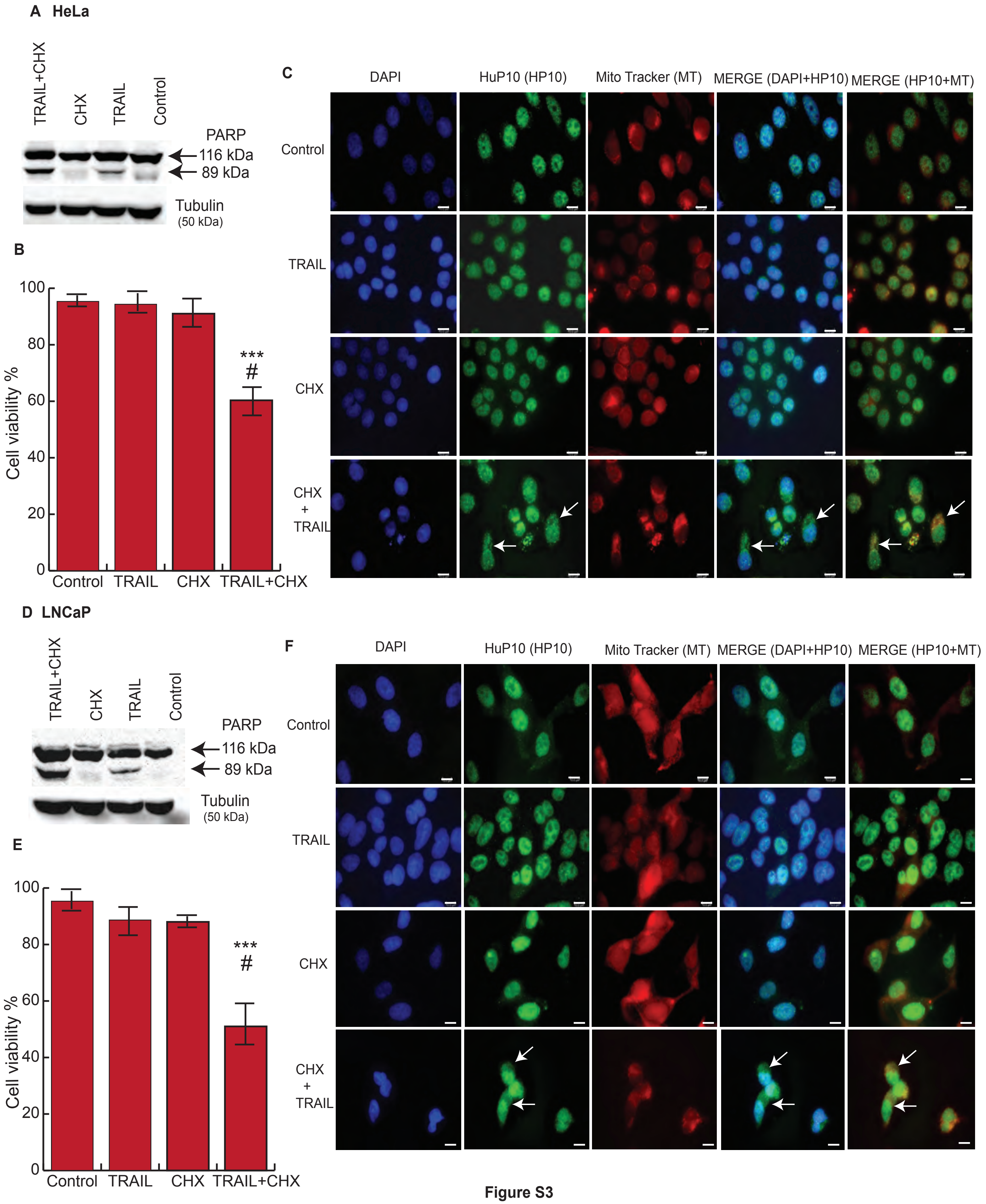
A HeLa
TRAIL+CHX
CHX
TRAIL
Control
PARP
116 kDa
89 kDa
Tubulin
(50 kDa)
B
Cell viability %
100
80
60
40
20
0
***
#
Control
TRAIL
CHX
TRAIL+CHX
C
DAPI
HuP10 (HP10)
Mito Tracker (MT)
MERGE (DAPI+HP10)
MERGE (HP10+MT)
Control
TRAIL
CHX
CHX + TRAIL
D LNCaP
TRAIL+CHX
CHX
TRAIL
Control
PARP
116 kDa
89 kDa
Tubulin
(50 kDa)
E
Cell viability %
100
80
60
40
20
0
***
#
Control
TRAIL
CHX
TRAIL+CHX
F
DAPI
HuP10 (HP10)
Mito Tracker (MT)
MERGE (DAPI+HP10)
MERGE (HP10+MT)
Control
TRAIL
CHX
CHX + TRAIL

**Figure S3**

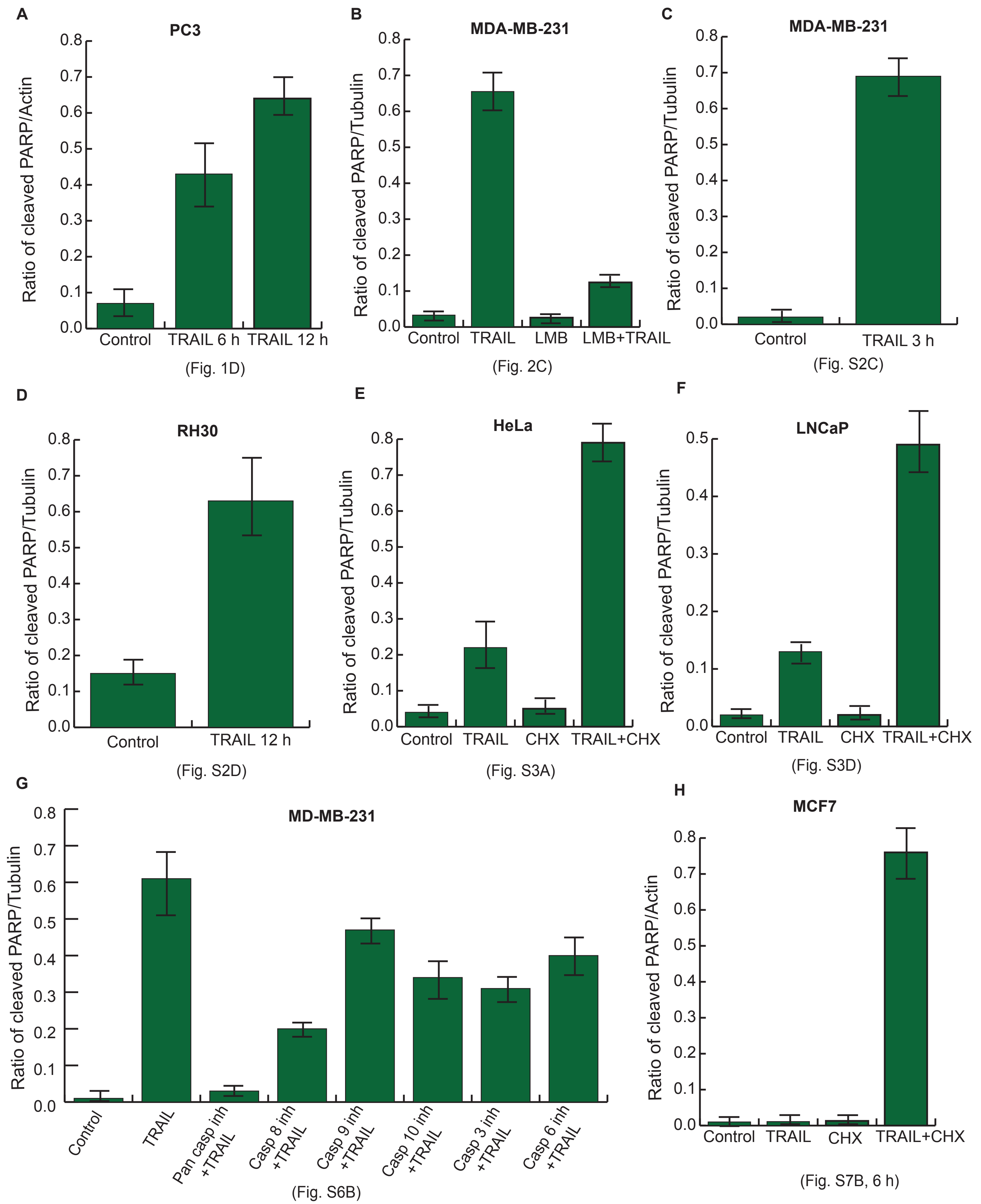
A
PC3
Ratio of cleaved PARP/Actin
0.8
0.7
0.6
0.5
0.4
0.3
0.2
0.1
0.0
Control
TRAIL 6 h
TRAIL 12 h
(Fig. 1D)
B
MDA-MB-231
Ratio of cleaved PARP/Tubulin
Control
TRAIL
LMB
LMB+TRAIL
(Fig. 2C)
C
MDA-MB-231
Ratio of cleaved PARP/Tubulin
Control
TRAIL 3 h
(Fig. S2C)
D
RH30
Ratio of cleaved PARP/Tubulin
Control
TRAIL 12 h
(Fig. S2D)
E
HeLa
Ratio of cleaved PARP/Tubulin
Control
TRAIL
CHX
TRAIL+CHX
(Fig. S3A)
F
LNCaP
Ratio of cleaved PARP/Tubulin
0.5
0.4
0.3
0.2
0.1
0.0
Control
TRAIL
CHX
TRAIL+CHX
(Fig. S3D)
G
MD-MB-231
Ratio of cleaved PARP/Tubulin
Control
TRAIL
Pan casp inh +TRAIL
Casp 8 inh +TRAIL
Casp 9 inh +TRAIL
Casp 10 inh +TRAIL
Casp 3 inh +TRAIL
Casp 6 inh +TRAIL
(Fig. S6B)
H
MCF7
Ratio of cleaved PARP/Actin
Control
TRAIL
CHX
TRAIL+CHX
(Fig. S7B, 6 h)

**Figure S4**

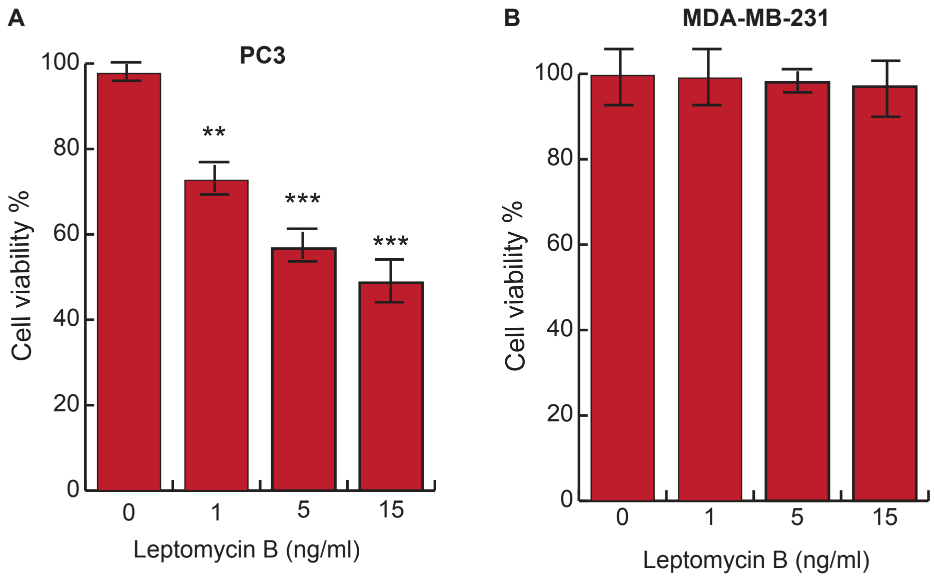
A
PC3
100
80
60
40
20
0
Cell viability %
**
***
***
0
1
5
15
Leptomycin B (ng/ml)
B
MDA-MB-231
100
80
60
40
20
0
Cell viability %
0
1
5
15
Leptomycin B (ng/ml)

**Figure S5**

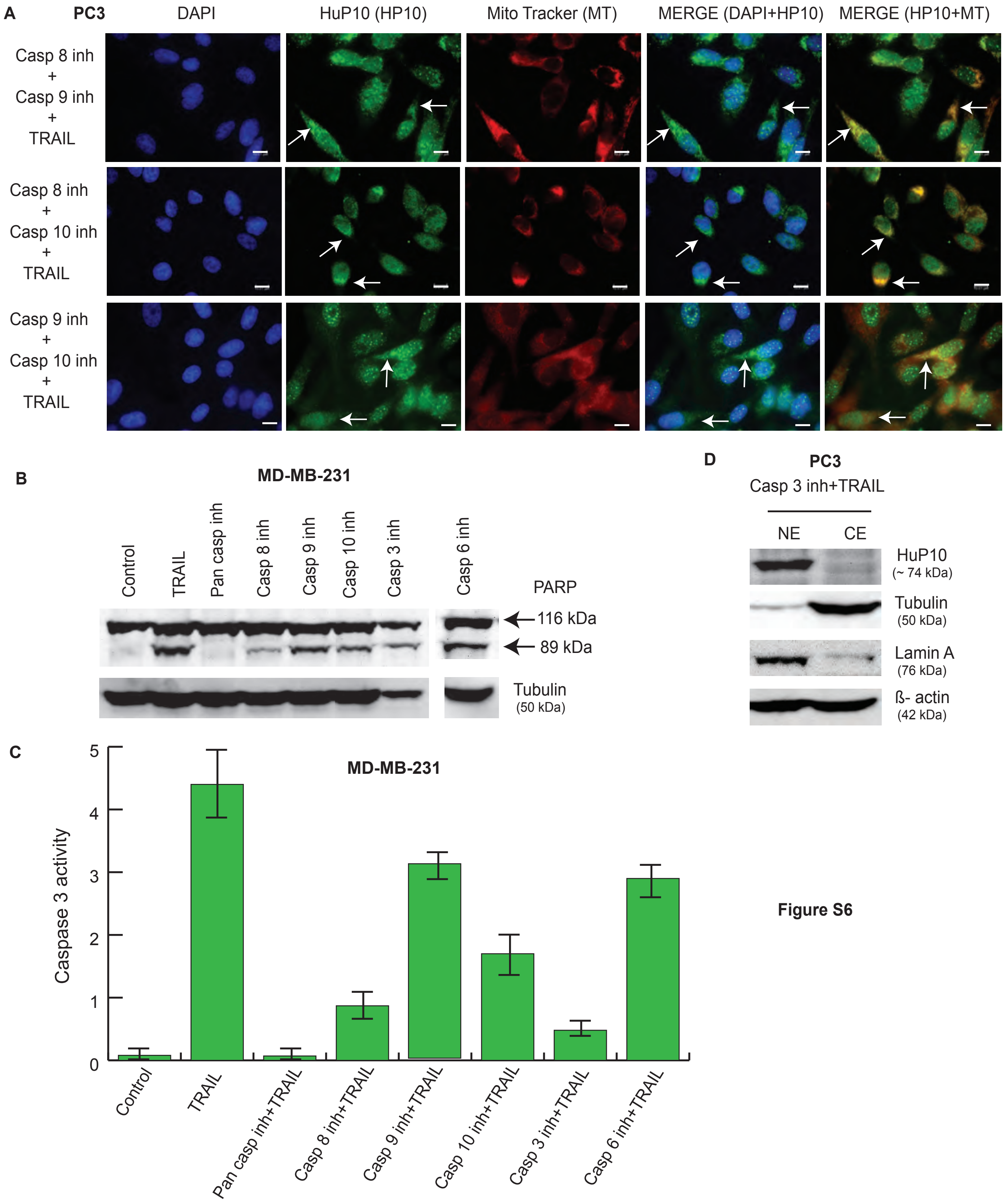
A
PC3
DAPI
HuP10 (HP10)
Mito Tracker (MT)
MERGE (DAPI+HP10)
MERGE (HP10+MT)
Casp 8 inh + Casp 9 inh + TRAIL
Casp 8 inh + Casp 10 inh + TRAIL
Casp 9 inh + Casp 10 inh + TRAIL
B
MD-MB-231
Control
TRAIL
Pan casp inh
Casp 8 inh
Casp 9 inh
Casp 10 inh
Casp 3 inh
Casp 6 inh
PARP
116 kDa
89 kDa
Tubulin
(50 kDa)
D
PC3
Casp 3 inh+TRAIL
NE
CE
HuP10
(~ 74 kDa)
Tubulin
(50 kDa)
Lamin A
(76 kDa)
ß- actin
(42 kDa)
C
MD-MB-231
Caspase 3 activity
0
1
2
3
4
5
Control
TRAIL
Pan casp inh+TRAIL
Casp 8 inh+TRAIL
Casp 9 inh+TRAIL
Casp 10 inh+TRAIL
Casp 3 inh+TRAIL
Casp 6 inh+TRAIL

**Figure S6**

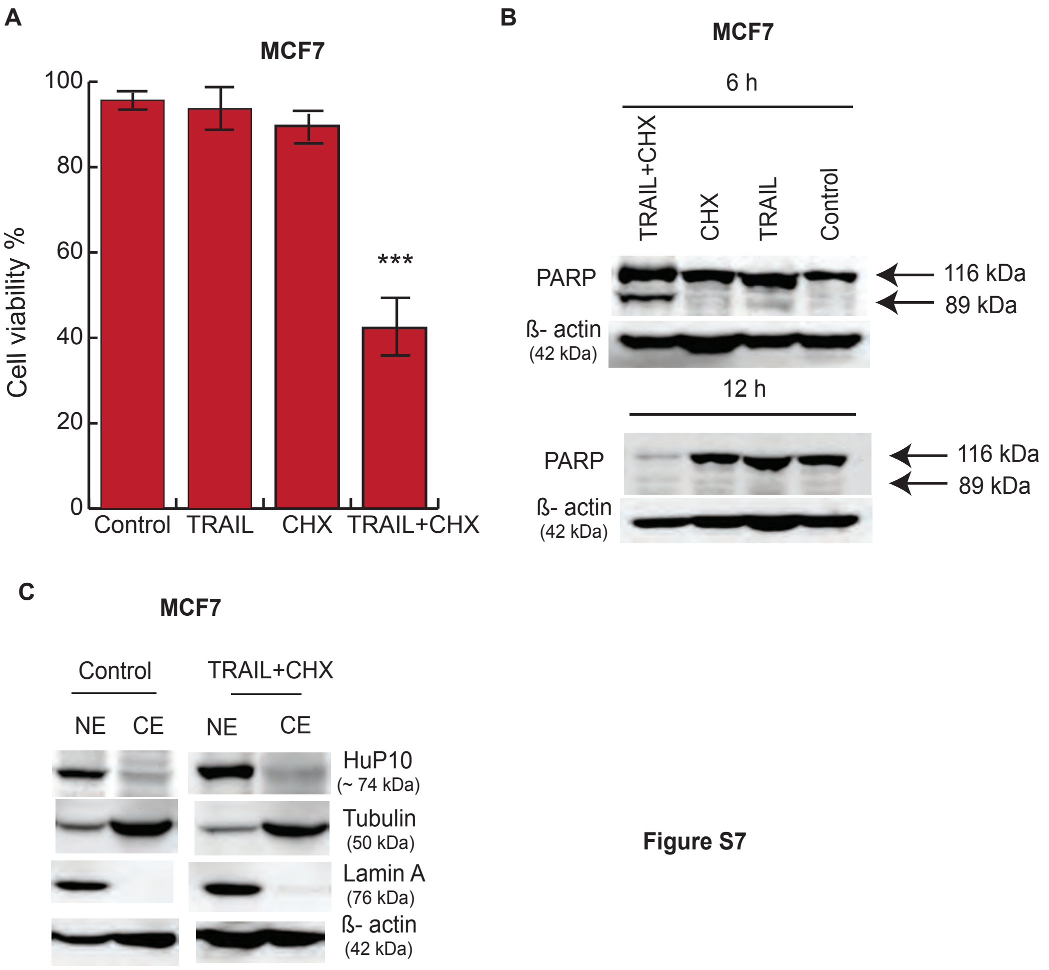
A
MCF7
Cell viability %
100
80
60
40
20
0
***
Control
TRAIL
CHX
TRAIL+CHX
B
MCF7
6 h
TRAIL+CHX
CHX
TRAIL
Control
PARP
116 kDa
89 kDa
ß- actin
(42 kDa)
12 h
PARP
116 kDa
89 kDa
ß- actin
(42 kDa)
C
MCF7
Control
TRAIL+CHX
NE
CE
NE
CE
HuP10
(~ 74 kDa)
Tubulin
(50 kDa)
Lamin A
(76 kDa)
ß- actin
(42 kDa)

**Figure S7**
